# Rice tiller number estimation based on an improved Swin-UNet model and multi-feature fusion

**DOI:** 10.3389/fpls.2025.1693548

**Published:** 2026-03-06

**Authors:** Xiao Liang, Junnuo Wu, Cheng Zhang, Lielie Qin, Xingcheng Liu, Yingli Cao

**Affiliations:** 1 Liaodong College, Dandong, China; 2 Shenyang Agricultural University, Shenyang, China; 3 Liaoning Rice Research Institute, Shenyang, China

**Keywords:** drone digital imaging, early-stage traits of rice, tillering characteristics, Swin-UNet model, particle swarm optimization, estimation of rice tillering number

## Abstract

Rice early tillering characteristics are key indicators for high-yield breeding, with tiller number and tillering rate as core parameters. High-throughput, temporal, and precise monitoring of tiller numbers via drone digital imagery provides quantitative support for tillering trait screening in breeding, serving as an important auxiliary tool for smart breeding. However, during the early tillering stage, complex backgrounds (e.g., water bodies, soil) and small, dense breeding plots pose challenges to high-throughput rice plant extraction and accurate tiller number estimation. To address this, this study proposes a rice tiller number estimation method based on an improved Swin-UNet model and multi-feature fusion. A PSO-optimized XGBoost model was constructed for tiller number estimation by integrating selected features. Experimental results show that the improved Swin-UNet model achieved a segmentation accuracy of 92.5% (7.2% higher than U-Net), and the PSO-XGBoost model, using 12 features (10 morphological and 2 color), yielded R²=0.85 and RMSE = 0.35. Application verification on 576 untrained breeding plots generated tiller number thematic maps, providing data support for germplasm tillering trait identification and advancing smart breeding.

## 1 Introduction

Rice is China’s primary grain crop, and high-yielding, high quality rice varieties screening is of great significance for ensuring food security. The number of effective panicles is one of the key factors influencing rice yield. The number of effective tillers determines the number of effective panicles and also has a significant impact on other yield-determining factors. Promoting the early initiation and growth of tillers is crucial for increasing rice yield (Li et al., 2003; Takai, 2024). Rice varieties that can rapidly produce a large number of tillers during the early tillering stage are referred to as early-tillering varieties. The number of tillers and the rate of tiller emergence are important parameters reflecting the early-tillering characteristics. The “early-tillering” trait has become a typical feature of modern high-yielding and efficient rice varieties (Liang et al., 2014). Accurate and high-throughput identification of early-tillering traits and variety screening are of critical importance. However, manual evaluation of early-maturing traits and calculation of tiller numbers in high-density breeding plots are time-consuming, labor-intensive, susceptible to subjective factors, and unable to provide long-term, quantitative monitoring of rice tiller status. These methods fail to meet the modern breeding industry’s demand for high-throughput, high-precision phenotypic data acquisition and cannot provide reliable quantitative support for breeding decisions. With the rapid development of unmanned aerial vehicle (UAV) remote sensing technology, its advantages of high-throughput and non-contact data collection have opened up new possibilities for estimating rice tiller numbers (Cao et al., 2024). This technology uses drones as a platform, equipped with multispectral cameras, high-resolution digital cameras, and other sensors, combined with intelligent decision-making software, to achieve real-time collection and analysis of farmland data. Its lightweight structure, flexible operation, and high spatio-temporal resolution enable rapid acquisition of key information such as crop growth status and pest and disease distribution. It can effectively observe and analyze important parameters such as crop leaf area index (LAI), relative chlorophyll content (SPAD), plant height, and tiller number, revealing their current growth status and future trends. For example, Qi et al. (2025) addressed the monitoring needs for wheat leaf area index (LAI) by extracting 12 vegetation indices and 8 texture features from drone multispectral images. After selecting optimal features through Pearson correlation analysis, they constructed a vegetation-texture composite feature using the recursive feature elimination method. Combining multiple linear regression, support vector regression, and gradient boosting regression algorithms, they developed an LAI estimation model. Jiang (2022) utilized UAV hyperspectral remote sensing technology to obtain rice canopy spectral data. By analyzing the spectral response characteristics of rice chlorophyll content, they employed machine learning methods to construct a chlorophyll content inversion model. Zhu et al. (2024) integrated unmanned aerial vehicle (UAV) multispectral vegetation indices with texture features, collected remote sensing imagery and ground-based measurement data during the tillering, flowering, and grain filling stages, extracted 15 vegetation indices and 35 texture features, screened sensitive features through correlation analysis, and employed three modeling strategies—spectral, texture, and integrated features—combined with four regression algorithms to conduct inversion modeling, thereby achieving precise estimation of chlorophyll content. Pei et al. (2017) utilized spectral information collected by a hyperspectral camera, integrating five key indicators of winter wheat—LAI, SPAD, crop nitrogen content, moisture content, and biomass—to construct a high-precision new model; Furthermore, Tao et al. (2020) also started from drone hyperspectral data to study the correlation between vegetation indices and growth detection indicators at different growth stages of winter wheat, successfully creating a growth condition detection map for winter wheat. Li et al. (2021) collected ground-based lidar and hyperspectral data, using two methods to estimate rice yield. The results confirmed that the validation accuracy of the linear yield model was significantly superior to that of the random forest yield model. Fan et al. (2025) addressed the need for estimating nitrogen content in rice grains by utilizing UAV hyperspectral remote sensing technology to obtain images during the jointing, flowering, and maturity stages. They automatically determined the optimal sensitive band width for the narrow-band normalized difference vegetation index (N-NDVI) using the inner rectangle method, established the correlation between plant growth and rice grain nitrogen content, and constructed an estimation model.

Traditional image segmentation methods first perform noise preprocessing on the image, then select appropriate image segmentation techniques based on features such as color and texture to segment the target and background, and finally use morphological algorithms to perform overall image restoration targeting issues in the image. Li et al. (2023) applied a Gaussian mixture model combined with a component of the Lab color space to segment the image background, achieving rapid segmentation of rice pixels in complex field environments. Ninomiya (2022) constructed decision trees using the CART algorithm and performed adaptive threshold adjustment to segment the growth prospects or backgrounds of different crops. Sun et al. (2025b) proposed a combined background segmentation scheme. This scheme adopts contrast enhancement filtering and integrates the excess green (ExG) feature value with the Otsu algorithm. It can effectively remove complex backgrounds in remote sensing images while completely preserving the morphological information of soybean seedlings, thereby providing a clear image foundation for subsequent crop-related analyses. Wu et al. (2014) improved the mean drift algorithm to smoothly extract color features from the target image. They then used the OTSU threshold segmentation method to distinguish between the target and background, with the innovation lying in the feature extraction aspect. Wang and Zhang (2020) completed the segmentation task of corn ears using the simple linear iterative clustering method and graph cut algorithm. Fan et al. (2015) extracted wheat texture and color features and employed the SVM learning method to precisely extract the wheat plant contour. Liu et al. (2014) combined the watershed algorithm with contour smoothing and Euclidean distance transformation to successfully separate corn ears and adhering grains. Traditional image segmentation methods can segment crop backgrounds, but the process is complex and slow, with each operational step significantly affecting the segmentation results. Changes in shooting angles and lighting conditions are also contributing factors to poor segmentation outcomes, and the methods lack robustness.

With the breakthrough progress made by convolutional neural networks (CNNs) in the field of image processing, they have become a hot topic of research in this area. Their core advantage lies in their powerful high-dimensional feature extraction capabilities, which provide critical technical support for image segmentation. Currently, researchers both domestically and internationally widely adopt deep convolutional neural networks for image feature extraction (Chua, 1997). This method utilizes an end-to-end learning mechanism, enabling image segmentation without the need for manually designed features, thereby effectively avoiding the limitations imposed by subjective feature selection in traditional methods. Wu et al. (2024) addressed the issues of high computational complexity and difficulty in mobile deployment of the DeepLabV3+ model by proposing a lightweight semantic segmentation algorithm that integrates the RepVGG and MobileViT modules. By introducing the SENet attention mechanism into RepVGG, the algorithm enhances global semantic feature extraction capabilities, significantly reducing computational costs compared to models such as FCN and PSPNet, while maintaining high accuracy and real-time performance in image segmentation at different flight altitudes. Lottes et al. (2020) designed a pixel-level semantic segmentation neural network model using encoding-decoding technology, specifically targeting the segmentation of weeds in farmland, to guide robots for precise removal. Sun et al. (2025a) further carried out in-depth research. They designed a dedicated feature extraction module, improved the original upsampling method of the model, and optimized the loss function, eventually proposing a lightweight field barnyard grass detection model. After conducting comparative tests between this model and the state-of-the-art models in the current field, it was found that the YOLOv8n-SSDW model among them exhibited outstanding comprehensive performance, providing a highly valuable technical reference and direction for the practical application scenarios of barnyard grass detection in paddy fields. Zhu et al. (2023) added a Shuffle Attention module to the backbone network of DeepLabV3+ to reduce semantic loss, enabling segmentation of mature rice plants and calculating the pixel proportion of rice to estimate rice growth density. In recent years, with the government’s increasing focus on smart agriculture and precision agriculture, many universities and research institutions have employed deep learning technology to address agricultural segmentation challenges. Xiong et al. (2017) captured rice images using a digital camera and developed a rice panicle segmentation scheme based on convolutional neural networks and superpixel segmentation. Although significant progress has been made in crop segmentation research within the field of deep learning, studies on the segmentation of rice plants in small-scale breeding plots with complex backgrounds remain in their infancy.

Since the mid-1990s, researchers both domestically and internationally have continuously incorporated the latest image processing technologies into studies on the tillering status of crops. Liu et al. (2025) addressed the issue of traditional threshing counting methods damaging the phenotypic structure of rice panicles by proposing a deep learning-based method for counting rice grains on panicles. They designed a deformable convolutional backbone network to extract rice panicle image features, constructed a feature correlation map between reference rice grains and panicle images through a feature correlation layer, and cascaded and reused multi-scale features to predict density distributions. Finally, they achieved counting by summing the density maps. Experimental results demonstrated that this method significantly outperforms existing benchmark methods in terms of counting accuracy and efficiency. Du et al. (2021) used visible light band remote sensing images to establish an inversion model for wheat tiller density. Experimental results demonstrate that the model, which uses vegetation indices and coverage as input parameters, has high feasibility in estimating wheat tiller density. Cao et al. (2020) selected a combination of sensitive wavelet characteristics and new spectral indices to construct a spectral detection model for the number of tillers in double-cropped rice. Wang et al. (2024) successfully developed a dynamic tillering analysis method for rice based on a dual Logistic model, which can provide precise and detailed dynamic information on rice tillering. Zhu et al. (2024) used unmanned aerial vehicle (UAV) multispectral imaging to obtain multispectral data during the rice tillering period. After image preprocessing, soil/vegetation endpoints were used to construct a spectral library, and employed fully constrained least squares method was employed for pixel decomposition. They established a regression model linking vegetation coverage to basic plant numbers, thereby improving the statistical accuracy of rice basic plant counts during the tillering stage and generating inversion maps to visually present distribution patterns, providing precise data support for field replanting and thinning operations. Yuan Fang et al. (Hayat et al., 2020) proposed an algorithm using hierarchical clustering to automatically calculate wheat tiller numbers in the field, which performed well in data with low plant density. Wan et al. (2020) combined multispectral images and RGB images captured by drones, extracted vegetation indices and texture features, and constructed a rice moisture content estimation model, achieving rapid and non-destructive detection of rice moisture content. Roth et al. (2020) processed RGB images obtained from repeated drone flights into multi-angle ground cover images to extract plant information and estimate tiller numbers. Piyanan et al. (2020) compared two rice varieties in terms of plant height, tiller numbers, and aboveground biomass using vegetation indices obtained from drones and ground-based non-destructive measurements as parameters, demonstrating the potential of drone remote sensing technology in assessing crop growth conditions.

Although existing research has made significant progress in the field of rice phenotyping analysis, technical challenges remain in addressing complex background segmentation and high-density tiller counting in small-scale breeding plots. Traditional manual feature-based segmentation methods have limitations in adapting to dynamic lighting conditions and background interference, and their operation processes are relatively complex, which may affect the efficiency of segmentation (Yu et al., 2024). Notably, in our specific research context (small-scale breeding plots with high-density tillers and complex backgrounds), mainstream models have shown certain adaptability limitations: SegFormer’s lightweight decoder makes a trade-off in fine-grained detail capture, posing challenges in distinguishing tightly adherent tiller boundaries in high-density scenarios; Swin-UNet variants rely on fixed window attention mechanisms, whose adaptability to scattered tiller distributions in small breeding plots needs improvement, and they often fail to fully integrate spectral features that are critical for resisting background interference; other Transformer-CNN fusion models (e.g., UNETR) mostly adopt simple feature concatenation, making it difficult to fully exploit the synergistic value between CNN-derived morphological features and Transformer-derived semantic features. To address these challenges, we propose a rice tiller number estimation model based on Swin-UNet and multi-feature fusion, aiming to overcome the challenges of plant segmentation in complex backgrounds and develop a rice tiller number estimation model suitable for high-density breeding plots, thereby providing technical support for high-throughput screening of early-maturing and fast-growing rice traits. The contributions and innovations of this paper are as follows:

1. Targeted multi-feature selection based on agronomic mechanisms: Combined with rice tillering agronomic principles (e.g., the correlation between leaf area and tillering ability, and the reflection of leaf color on nutritional status), 12 tillering features (10 morphological features and 2 color features) are systematically selected. This selection not only leverages the joint feature extraction advantages of deep learning but also further screens and optimizes feature combinations based on agronomic logic, ensuring the rationality and effectiveness of input features for the estimation model.
2. Optimized tiller number estimation model: The Particle Swarm Optimization (PSO) algorithm is used to optimize the hyperparameters of the XGBoost model, constructing a PSO-XGBoost tiller number estimation model. This model fully utilizes the nonlinear fitting ability of XGBoost and the efficient parameter optimization ability of PSO, effectively improving the estimation accuracy of rice tiller number in high-density breeding plots.
3. Practical application-oriented technical chain: A complete technical process from drone image acquisition, image preprocessing, rice plant segmentation, feature extraction, to tiller number estimation and thematic mapping is formed. The model was validated using breeding test plot data collected in 2024, and thematic maps of tiller number distribution were generated, providing direct and quantitative data support for the identification of tiller traits in rice germplasm resources.

## 2 Materials and methods

### 2.1 Data collection

The study was conducted in June and July 2023 and 2024 at the experimental site of the Liaoning Province Rice Research Institute. The experimental site covers an area of 3.3 acres, with 1,404 breeding plots, each plot consisting of 3 rows × 9 holes, totaling approximately 1.08 m² (1.2 m × 0.9 m). The region has a temperate continental semi-humid monsoon climate, with an annual average temperature of 8 °C, annual precipitation of 659.6 mm, a frost-free period of 147 days, and annual sunshine hours of 2,527 hours. The rice plants exhibit diverse morphological characteristics, meeting experimental requirements, and the well-organized rice fields are suitable for drone data collection.

**Figure 1:**
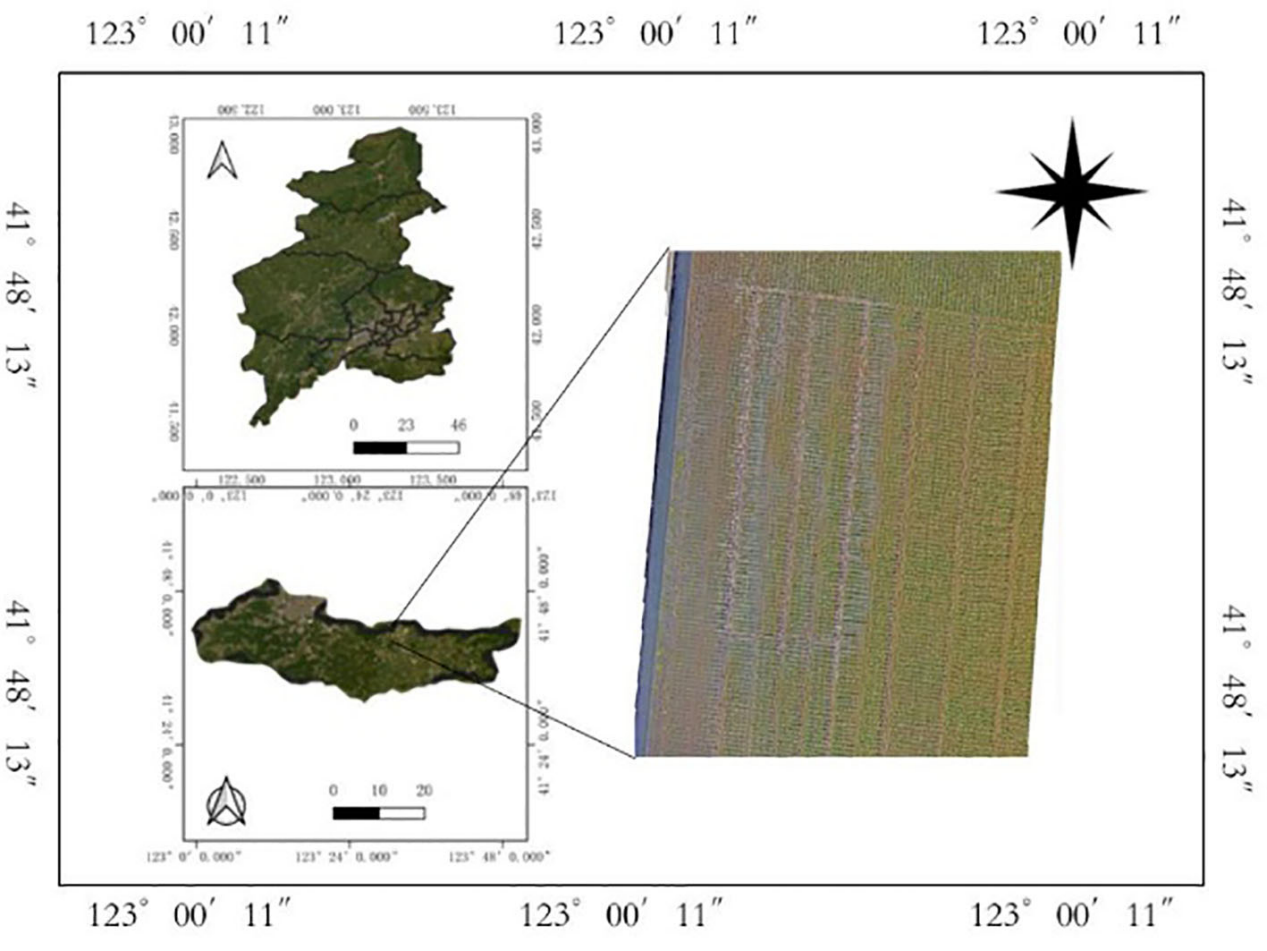
Overview of research area.

From June to the end of July in both 2023 and 2024, we synchronously collected ground-based actual tiller count data and UAV digital imagery data for rice at different tillering stages (**Figure 1**). In 2023, 120 breeding plots were selected, and in 2024, 200 breeding plots were selected. Four rice plants were selected from each plot to collect the actual tiller counts. The collection periods and data volumes are shown in **Table 1**, and some of the actual tiller data are shown in **Table 2**.

**Table 1:** Tillering data test collection time.

Test date	Tillering period	Actual tillering data collected
June 21, 2023	Early tillering	120*4 holes
June 30, 2023	Mid-tillering	
July 7, 2023	Late tillering	
June 15, 2024	Early tillering	200*4 holes

**Table 2:** Schematic illustration of part tillering data in the field.

Sampling point number	Breeding plot number												
	H1	H2	H3	H4	H372	H373	H374	H375	H793	H793	H793	H793
1	2	3	2	3	7	13	11	11	4	7	4	8
2	2	4	3	3	8	11	12	13	4	7	3	6
3	3	2	2	4	9	9	10	9	3	9	4	8
4	3	3	2	2	9	9	11	10	5	8	4	7

**Figure 2:**
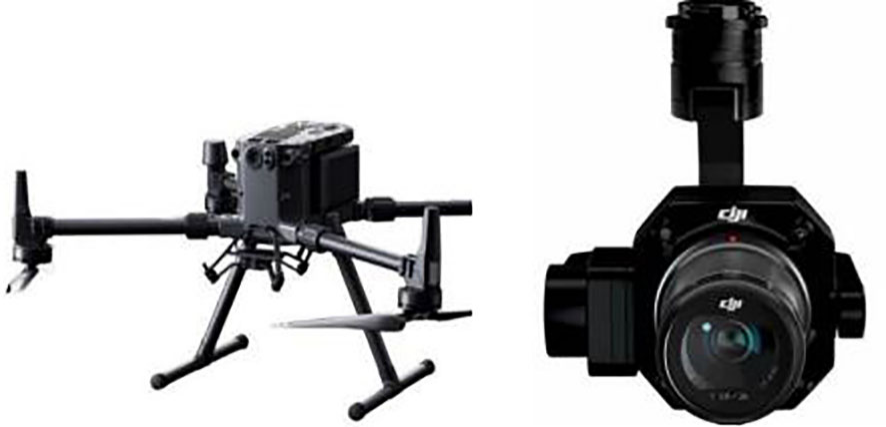
Image acquisition equipment.

Digital image data collection for rice drones uses a DJI M300 RTK drone equipped with a Zenmuse P1 camera (**Figure 2**), with 45 million effective pixels, a flight altitude of 12 m (0.15 cm/pixel), a zigzag flight path, a lateral overlap rate of 70%, a longitudinal overlap rate of 80%, and fixed-point orthophoto flight.

**Figure 3:**
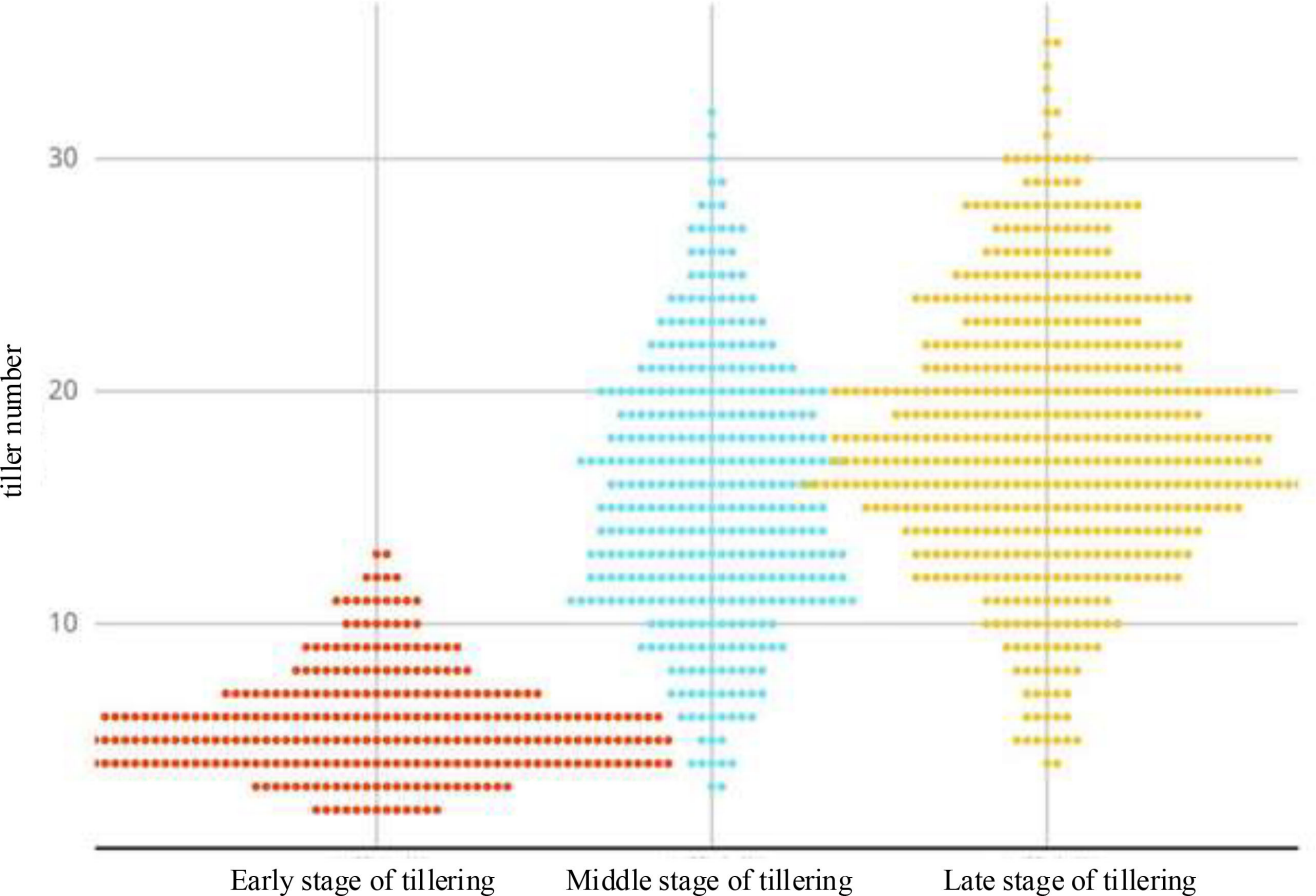
Tiller number truth distribution map.

The number of tillers in each breeding plot was counted separately. Considering that the true values of the tiller data collected on June 15, 2024, were very close to those of the first collection in 2023, the data from these two collections were merged for statistical analysis. The distribution of tiller numbers at different collection times is shown in **Figure 3**.

From this statistical chart, it can be seen that the number of tillers during the early tillering stage of rice ranges from 2 to 12 tillers per hill. As the rice grows and develops, the number of tillers during the middle tillering stage increases rapidly, reaching a maximum of 30 tillers per hill, with the number of tillers peaking during the late tillering stage. The number of tillers in rice varies with the growth and development of the plant.

### 2.2 Data preprocessing

Image preprocessing is a key step in improving image quality and removing interference. In the collection of rice canopy images, environmental and technical limitations often lead to problems such as uneven brightness, noise, and color tone deviations, which affect tillering period analysis. Therefore, it is necessary to enhance the original images, such as adjusting contrast, curves, and color saturation, to extract key information. Typically, a combination of multiple enhancement methods is used to achieve the best results.

#### 2.2.1 Histogram equalization

Considering the characteristics of visible-light images in rice tillering stage (low contrast, blurred plant-background boundaries, and uneven illumination caused by drone shooting angles), Adaptive Histogram Equalization (CLAHE) was selected for image enhancement (instead of global histogram equalization, which easily over-enhances noise in uniform areas). This method divides the image into grid cells for histogram equalization. effectively enhancing local details while avoiding over-saturation of bright areas.

The specific implementation parameters were set as follows:

Grid cell size: 8×8 (determined by testing 4×4, 8×8, and 16×16 grids; 8×8 achieved the best balance between detail enhancement and computational efficiency).
Clip limit (contrast threshold): 2.0 (limits the maximum pixel value in the local histogram to suppress noise amplification; values >2.0 led to excessive enhancement of soil texture interference, while values<1.5 failed to improve leaf edge clarity).
Interpolation method: Bilinear interpolation (used to eliminate block artifacts caused by local equalization, ensuring smooth transitions between adjacent grid cells).

**Figure 4:**
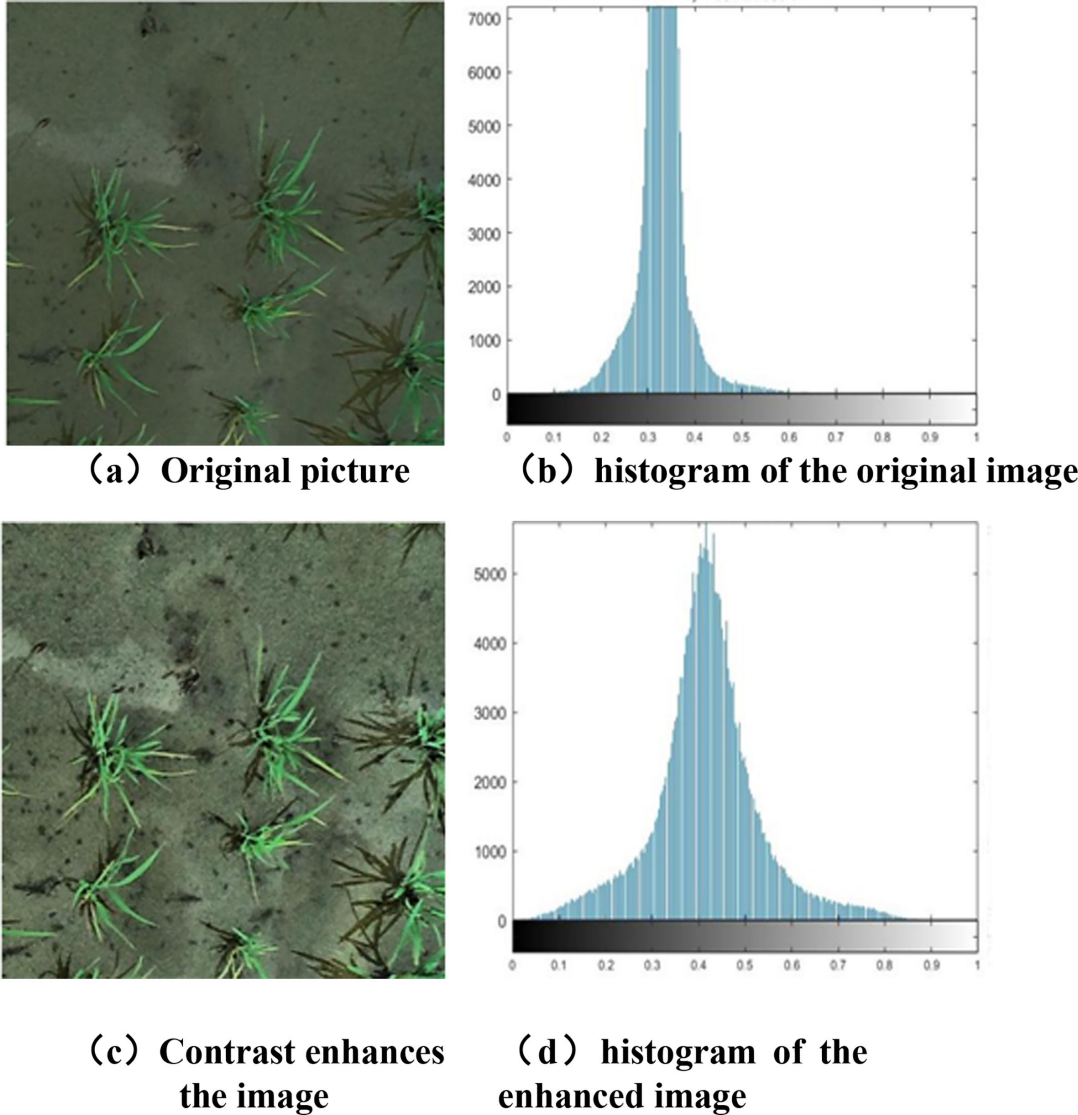
Contrast enhancement before and after contrast. **(a)** original picture, **(b)** histogram of the original image, **(c)** contrast enhances the image, **(d)** histogram of the enhanced image.

**Figure 4** shows the effects of contrast enhancement (both before and after) on rice tillering stage images and their corresponding histograms, which can be analyzed from visual performance and histogram characteristics. The original image (a) is overall dark, with low contrast between plants and the background, blurred boundaries, and unclear details such as leaf morphology. After enhancement, the plants stand out more, the boundaries are clear, the overall brightness and contrast are significantly improved, and the detail distinguishability is greatly enhanced. Histograms reflect an image’s gray-level distribution: the original image’s histogram has gray levels concentrated in a narrow range and a high, steep peak, with pixel gray levels clustered and few gray-level layers, resulting in low contrast. In contrast, the enhanced image’s histogram has more dispersed gray-level distribution, covers a wider range, and has a wide, low peak; as it utilizes more gray-level layers, the contrast is enhanced.

#### 2.2.2 Filter noise reduction

Visible - light images of the rice tillering stage captured by unmanned aerial vehicles (UAVs) are prone to salt - and - pepper noise interference. Such noise damages key features like tiller branch edges and small tiller textures, thereby affecting the segmentation accuracy of the subsequent Swin - UNet model. In this study, median filtering is adopted for noise removal. This method replaces the gray value of the central pixel with the median value of pixel gray values within the filtering window, which can effectively eliminate salt - and - pepper noise while maximally preserving spatial features such as plant edges and tiller details.

The specific implementation steps and parameter settings are as follows:

Determination of window size: The optimal parameters are determined by comparing the processing effects of three commonly used window sizes: 2×2, 3×3, and 5×5:

2×2 window: Only about 60% of the salt - and - pepper noise can be removed, and the processing of dense noise areas is incomplete.
5×5 window: Although more than 95% of the salt - and - pepper noise can be removed, it will lead to texture loss of small tillers (with a width of 0.5–2 cm) and blurring of leaf edges.
Finally, a 3×3 window is selected: It can remove 92% of the salt - and - pepper noise and completely retain key features such as tiller branch edges and leaf veins, causing the least interference to subsequent feature extraction.

Edge processing strategy: The “symmetric padding” method is used to process the edge pixels of the image (that is, the virtual pixels outside the image boundary are mirrored and copied to supplement the edge pixel information required by the filtering window), avoiding the gray value distortion of the image edge area caused by traditional “zero padding” and ensuring the integrity of rice plants at the image edge.

**Figure 5:**
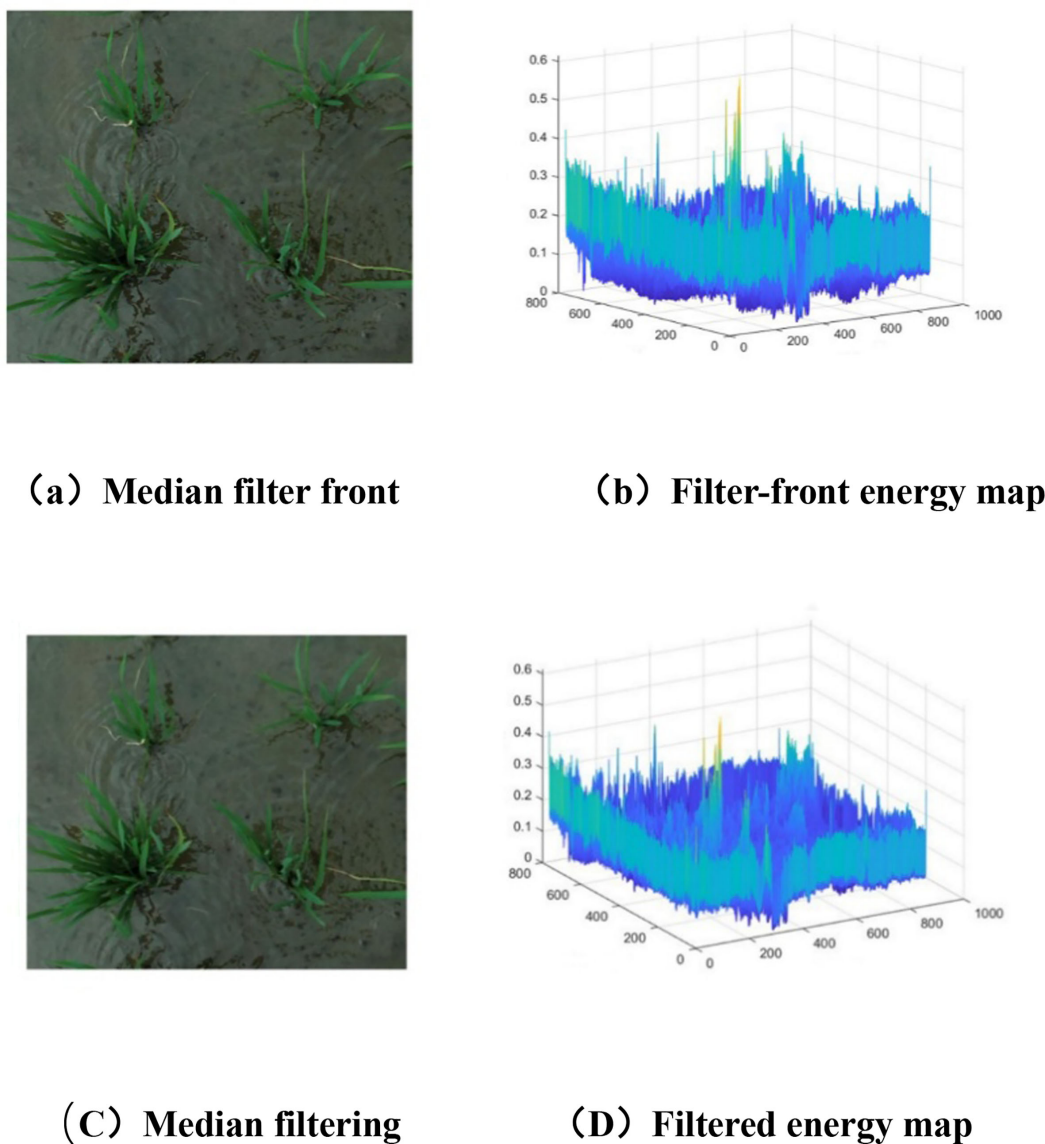
Comparison before and after filtering. **(a)** median filter font, **(b)** filter-font energy map, **(C)** median filtering, **(D)** filtered energy map.

**Figure 5** shows the comparison of effects before and after median filtering, which can be analyzed from two aspects: visual performance and energy map characteristics. Before filtering (Figure (A)), there are obvious salt - and - pepper noises in the image, with scattered white and black spots distributed on the soil surface and the edges of rice leaves, interfering with the boundary judgment between rice leaves and the background, and details like leaf contours and small tiller textures are also unclear. The corresponding energy map (Figure (B)), as a three - dimensional representation based on the gray value distribution of the image, has a large number of “spike” interferences in the energy distribution; these spikes correspond to the high - energy outliers of salt - and - pepper noise, masking the true texture energy features of rice leaves and soil themselves and causing effective features to be overwhelmed by noise. After median filtering with a 3×3 window, in the filtered image (Figure (C)), the edges of rice leaves are smoother, the noise points in the soil background are greatly reduced, and the morphological features of rice such as tillers and leaves are clearer, with key spatial details retained. Meanwhile, in the filtered energy map (Figure (D)), the spikes caused by noise are effectively suppressed, and the energy distribution is smoother and more in line with the true texture fluctuation laws of rice leaves and soil, intuitively reflecting the balanced effect of median filtering in “removing noise” while successfully preserving the key texture features of crops.

### 2.3 Dataset construction and evaluation metrics

#### 2.3.1 Dataset construction

To achieve precise segmentation of rice plants, the open-source annotation software Labelme was used to accurately annotate the images. The polygon lasso tool was used to manually draw the rice plant areas, ensuring accurate boundaries. Ultimately, a dataset containing 560 images and 13,340 annotated points was created, and a JSON format label file was generated, which was then parsed into a PNG semantic label map for model training.

**Figure 6:**
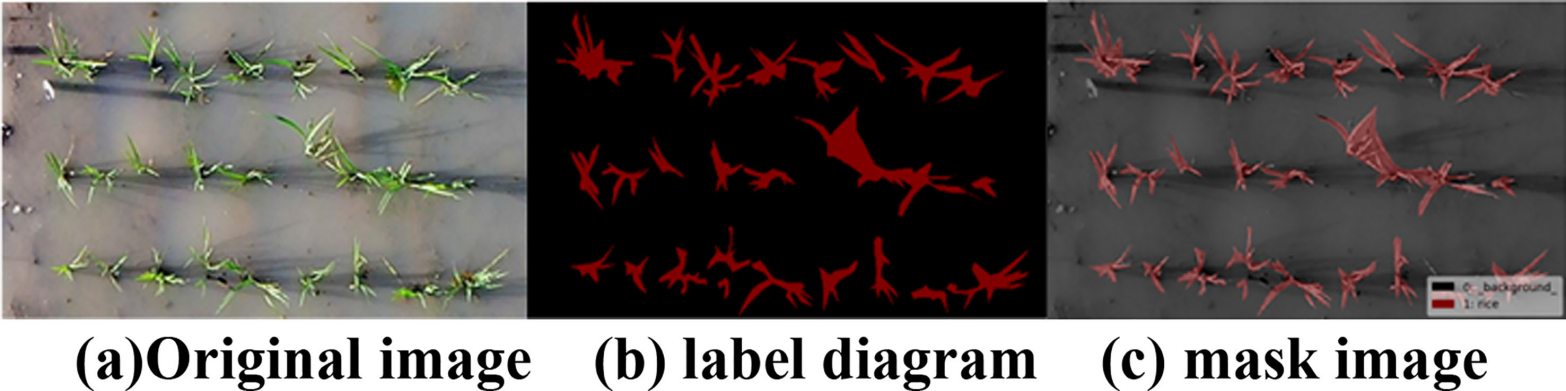
Image annotation and visualization. **(a)** original image, **(b)** label diagram, **(c)** mask image.

After analysis, labeled images are obtained, with different pixel values corresponding to different categories. Based on research requirements, the images are divided into two categories: rice plants and background. The pixel values are 0 and 1, where 0 represents the background and 1 represents the rice plant, denoted as “rice.” By converting the pixel colors, a colored labeled image is obtained, allowing the category information of the labeled image to be directly identified. **Figure 6** illustrates the visualized labels and mask images before and after image annotation.

#### 2.3.2 Segmentation model evaluation indicators

The commonly used evaluation metrics for image segmentation are the mean intersection-over-union (mIoU), which represents the ratio of the intersection and union of the actual segmentation results and the label map. Pixel accuracy (PA) is the percentage of correctly estimated pixels out of the total estimated pixels. mIoU is the average intersection-over-union for each category, and is calculated using the following Equations 1, 2:

(1) $$PA=\frac{TP+TN}{TP+FP+FN+TN}$$

(2) $$mIoU=\frac{1}{c}\sum_{1}^{c}\frac{TP_{i}}{TP_{i}+FP_{i}+FN_{i}}$$

In the formula, the higher the pixel accuracy ratio and the average intersection-over-union ratio coefficient, the higher the segmentation accuracy. Here, T/F denotes the correctness of the estimation results, and P/N denotes the estimation results, where TP represents the total number of pixels correctly estimated as target rice, FP represents the total number of pixels incorrectly estimated as background, TN represents the total number of pixels correctly estimated as background, and FN represents the total number of pixels incorrectly estimated as target rice.

#### 2.3.3 Evaluation criteria for tiller number estimation models

When validating the model’s effectiveness during the tillering stage, 80% of the samples and the true tillering values were randomly selected to train the model, while the remaining 20% of the samples were used to test the model’s performance and accuracy. To assess the model’s stability across various metrics, the following measures were employed: coefficient of determination (R²; Equation 3), root mean square error (RMSE; Equation 4), normalized root mean square error (nRMSE; Equation 5), and mean absolute error (MAE; Equation 6). The formulas for these metrics are as follows:

(3) $$R^{2}=\frac{\sum_{i=1}^{n}{\left(\hat{y}-\bar{y}\right)}^{2}}{\sum_{i=1}^{n}{\left(y_{i}-\bar{y}\right)}^{2}}$$

(4) $$RMSE=\sqrt{\frac{1}{n}\sum_{i=1}^{n}{\left(y_{i}-{\hat{y}}_{i}\right)}^{2}}$$

(5) $$nRMSE=\frac{\sqrt{\frac{1}{n}\sum_{i=1}^{n}\left(y_{i}-{\hat{y}}_{i}\right)}}{N_{F}}\times 100\%$$

(6) $$MAE=\frac{1}{m}\sum_{i=1}^{m}\left|\left(y_{i}-{\hat{y}}_{i}\right)\right|$$

### 2.4 Preliminary experiment on rice plant segmentation based on OTSU and UNet

#### 2.4.1 Rice plant segmentation based on OTSU

Crown images of rice obtained during the tillering stage have complex backgrounds, including soil, water bodies, and reflections of rice plants, which introduce interference. Additionally, there is a significant number of algae distributed around the roots of rice plants, and their proximity to the plants can lead to misclassification. Furthermore, during the early stages of tillering, rice plants are relatively small, and issues such as poor lighting or yellowing leaves due to poor growth conditions further complicate the segmentation of rice crown images. In this study, traditional image segmentation methods were validated for these scenarios.

The color difference between rice images and the background is significant, and color components can serve as a breakthrough point for segmentation. To distinguish green plants from the land background, the green signal weight can be enhanced to improve contrast. The principle involves using vegetation indices to strengthen the green channel, enhancing the contrast between green plants and the background to achieve effective segmentation. The experiment employed the ExG (Extra Green) color feature and the OTSU threshold segmentation algorithm for rice plant segmentation, and is calculated using the following Equation 7.

(7) EXG=2G−R−B

In this process, R, G, and B represent the actual values of pixels in the image. The process of grayscaling the color rice image using the ultra-green color feature is as follows.

The ExG-processed image highlights the green information of the rice canopy. To distinguish between the background and the canopy, the OTSU thresholding algorithm is used to binarize the grayscale image, generating a binary image. To verify the segmentation effect of the maximum interclass variance method, the coordinates of the white pixels in the binary image are mapped to the original RGB image.

**Figure 7:**
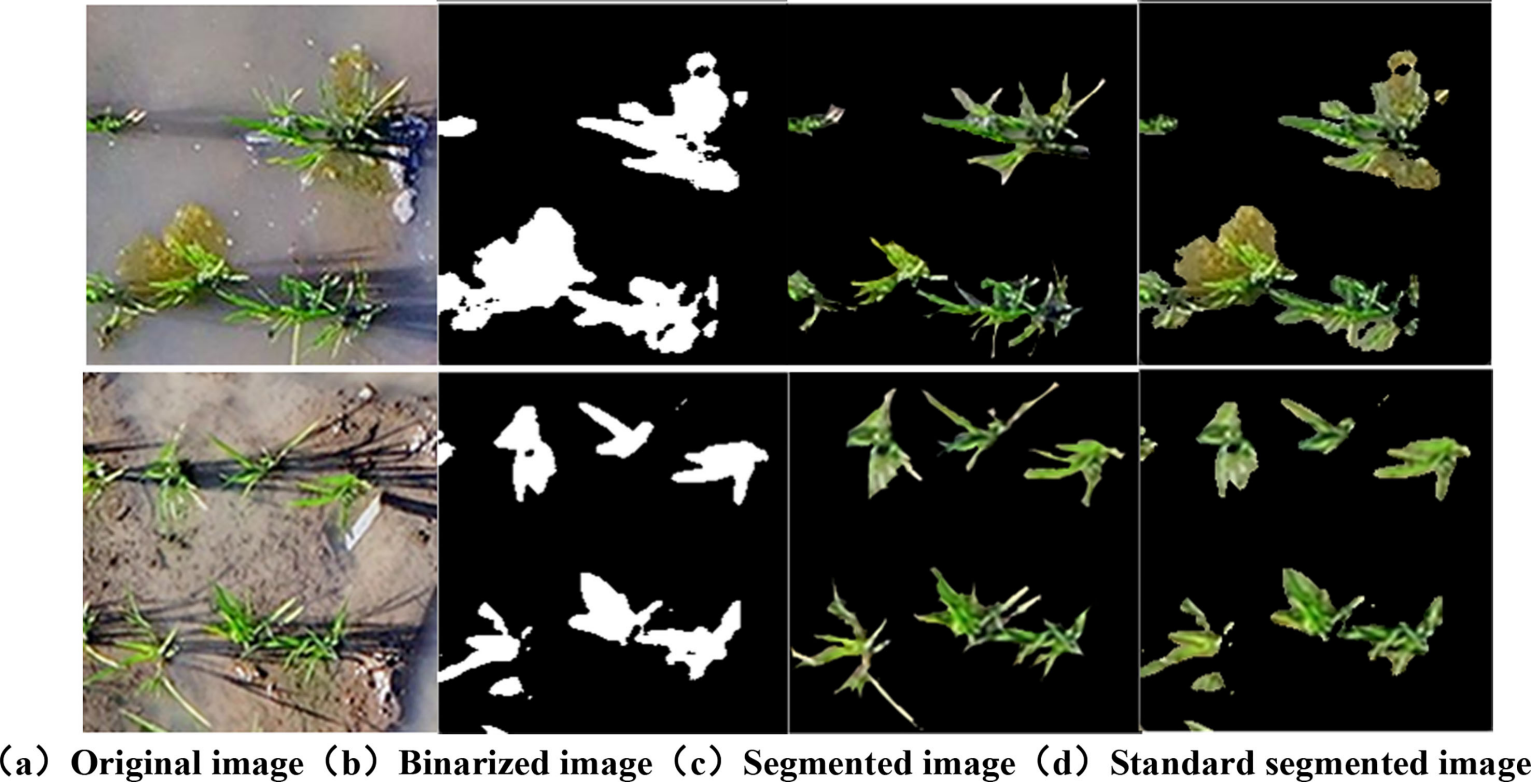
Rendering of OTSU segmentation with different color components. **(a)** original image, **(b)** binarized image, **(c)** segmented image, **(d)** standard segmented image.

As shown in **Figure 7**, the OTSU threshold segmentation algorithm can effectively separate rice from the background in grayscale images and remove interference such as soil, reflections, and water bodies, proving its effectiveness on the experimental data. However, it still has limitations in complex breeding plot backgrounds.

#### 2.4.2 Rice plant segmentation based on U-Net

The U-Net network architecture, specifically the Encoder-Decoder structure, is a widely adopted fully convolutional neural network (FCNN) technique. The Encoder component is responsible for feature extraction and consists of two 3×3 convolutional layers and a 2×2 max pooling layer, which perform downsampling. The Decoder component handles upsampling and comprises an upsampling convolutional layer, feature concatenation, and two additional 3×3 convolutional layers, enabling the upsampling process. The Encoder component is used to capture contextual information in the image, while the Decoder component is used to precisely locate details within the image. This is particularly important for segmenting rice images, as it is necessary to distinguish between rice plants and the background while also accurately delineating the contours of each rice plant to achieve pixel-level segmentation.

The U-Net segmentation model has achieved significant results in the processing of remote sensing images in the past, but it still has shortcomings: (1) It lacks global semantic features, has poor ability to extract detailed information, and is inaccurate in identifying boundary information, which can easily lead to classification errors. (2) It cannot express multi-scale information for small-sized plants of the same category but different sizes. To address these issues and drawing inspiration from the network structures of current state-of-the-art models, we propose a parallel architecture combining Swin Transformer and U-Net. This architecture separately collects global semantic information and shallow spatial information from feature maps, then fuses these different feature maps to incorporate richer feature information.

### 2.5 Improvements to segmentation models using Swin transformer

U-Net faces challenges in remote sensing image processing, including insufficient global semantic features, inaccurate boundary identification, and limited multi-scale representation capabilities. To address these issues, this paper combines the Swin Transformer with U-Net while retaining U-Net’s symmetric structure (Cao et al., 2022; Yue et al., 2024; Sun et al., 2025a). To address the issue of low classification accuracy for rice boundaries in drone remote sensing images, the Swin Transformer structure is integrated into the encoder to establish long-range dependencies, enabling the model to understand remote sensing images from a global perspective and thereby reduce misclassification of boundary pixels; To address the poor recognition performance of small objects, sub-pixel convolutions are used in the decoder to improve the upsampling structure, enabling thorough contextual feature fusion with feature maps from all layers in the encoder. This allows the model to retain as much image detail as possible, thereby enhancing its recognition of small objects.

#### 2.5.1 Swin-Unet network structure design

**Figure 8:**
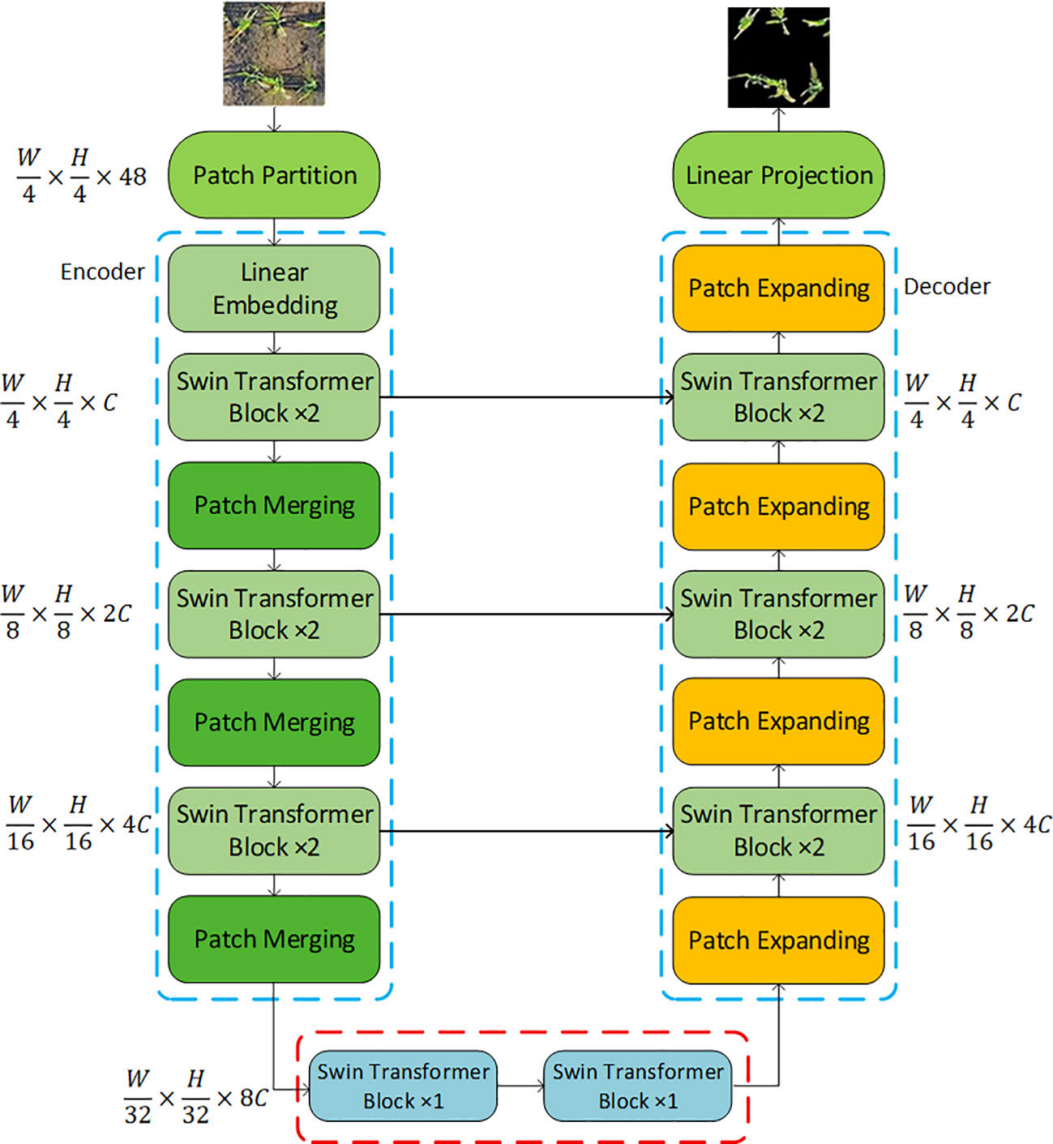
Network structure diagram.

Swin-Unet consists of an encoder, a bottleneck layer, a decoder, and skip connections. The encoder converts features into sequences by dividing rice images into 4×4 patches and applying linear embeddings. Hierarchical feature representations are generated through multiple Swin Transformer modules and patch merging layers. The decoder consists of Swin Transformer modules and patch expansion layers, which utilize skip connections to fuse multi-scale features from the encoder and compensate for spatial information loss caused by downsampling. The patch expansion layers are used for upsampling, ultimately restoring the input resolution and outputting pixel-level segmentation predictions (Cao et al., 2022). The overall network structure is shown in **Figure 8**.

#### 2.5.2 Swin-Unet network module

**Figure 9:**
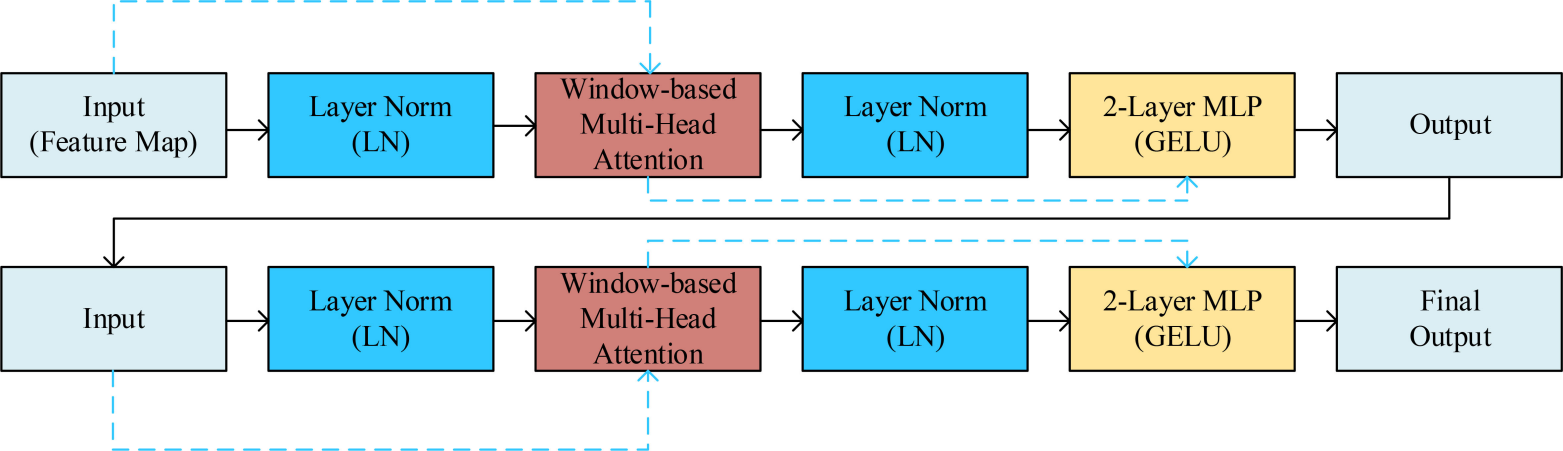
Swin Transformer block.

1. Swin Transformer: A Transformer-based image classification model that addresses computational and memory overhead issues in processing large-sized images through a sliding window mechanism. Its innovative features include: 1) a windowed self-attention mechanism that transforms global attention into local attention, reducing computational costs; 2) a hierarchical structure that enables cross-level information transfer through window segmentation; 3) the use of transposed convolutions to replace fully connected layers, reducing parameters and computational complexity. Swin Transformer demonstrates outstanding performance in image classification, object detection, and semantic segmentation. Its modules employ window self-attention (W-MSA) and shifted window self-attention (SW-MSA), with each module comprising Layer Norm (LN), multi-head self-attention, residual connections, and two layers of MLP with GELU activation (Cao et al., 2022). **Figure 9** illustrates two consecutive Swin Transformer modules.
2. Encoder: The encoder component primarily consists of an input layer, a Swin Transformer, and a patch merging layer. Its main function is to abstract and encode the input data, providing more useful information for subsequent decoding operations.
3. Decoder: The decoder primarily consists of a Swin Transformer and a patch expansion layer. Its main function is to fuse the contextual features extracted by the encoder with multi-scale features. The patch expansion layer primarily merges feature maps from adjacent dimensions into feature maps with twice the upsampled resolution, thereby extracting high-resolution information.
4. Optimization function Lion: Compared to the AdamW optimizer, the Lion optimizer does not perform square root, division, or square root operations, and caches one fewer set of second-order moments parameters than the AdamW optimizer. This results in faster training speeds while also saving memory.

### 2.6 Application of Swin-UNet for plant segmentation and post-processing

The Swin-UNet model improves the accuracy of rice plant segmentation through global semantic perception, but the segmentation results still suffer from issues such as local adhesion and noise interference. To further optimize segmentation quality, connected component labeling is used to identify independent plant regions and remove small-area noise, ensuring the validity of subsequent analysis. To address the issue of leaf adhesion caused by dense plant growth during the tillering stage, we employed distance transformation and a cyclic threshold segmentation algorithm to separate adhered regions into individual plant images. This ensures the accurate extraction of morphological features (such as plant area, perimeter, and bounding rectangle) and color features (such as greenness and vegetation coverage).

#### 2.6.1 Connected domain mark

**Figure 10:**
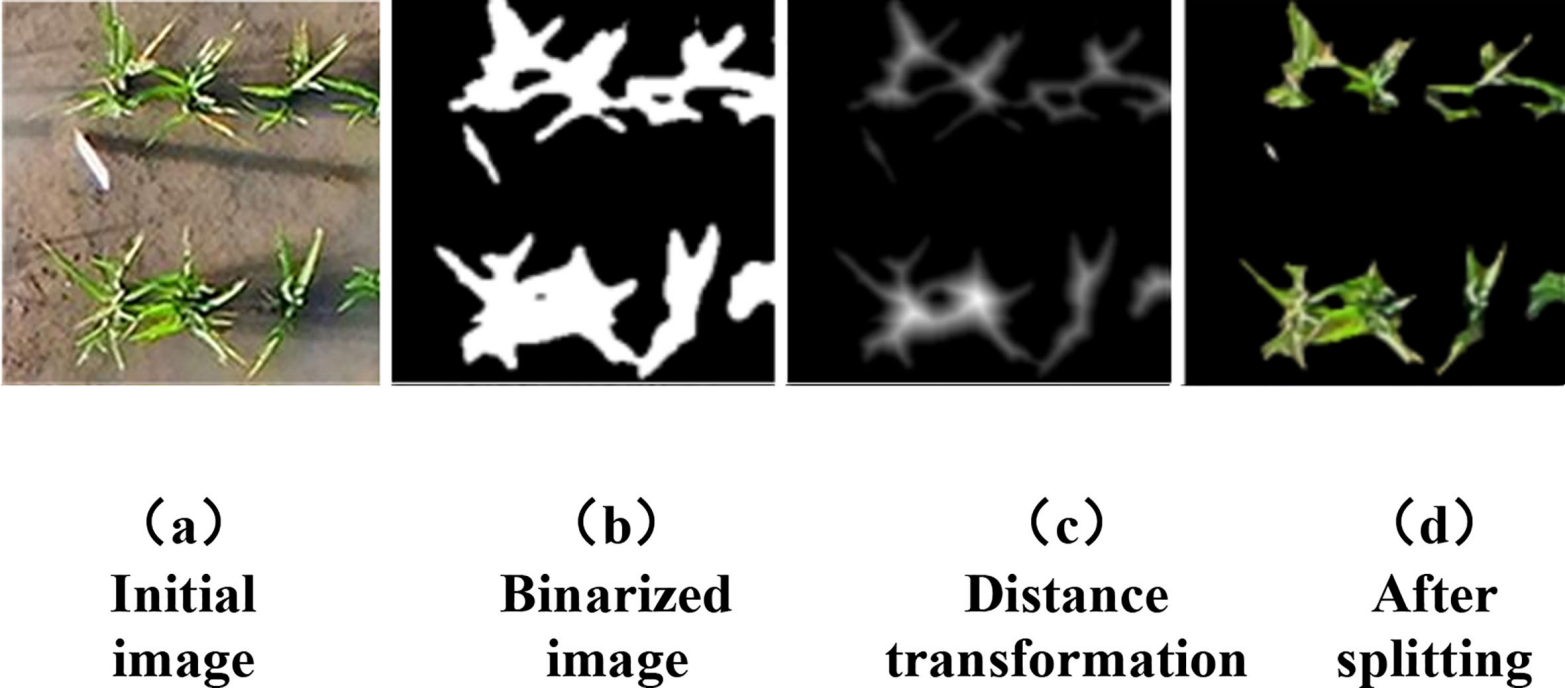
Pixel area extraction plot. **(a)** initial image, **(b)** binarized image, **(c)** distance transformation, **(d)** after splitting.

The binary image of rice plants contains single plants and overlapping plants. During the early stage of tillering, there is little overlap, but in the middle and late stages, plant growth accelerates, increasing the difficulty of segmentation. The primary task is to separate individual rice plants in single holes. The ‘bwconncomp()’ function in MATLAB is used to analyze pixel values and identify connected regions. As shown in **Figure 10**, some connected regions (red matrix) may contain multiple overlapping rice plants.

After obtaining each target connected region, small-area noise is first removed, followed by characteristic analysis and feature extraction. Edge points are generated by extracting boundary contours, and the minimum bounding matrix is solved using the rotation snap algorithm.

#### 2.6.2 Adhesion area division

**Figure 11:**
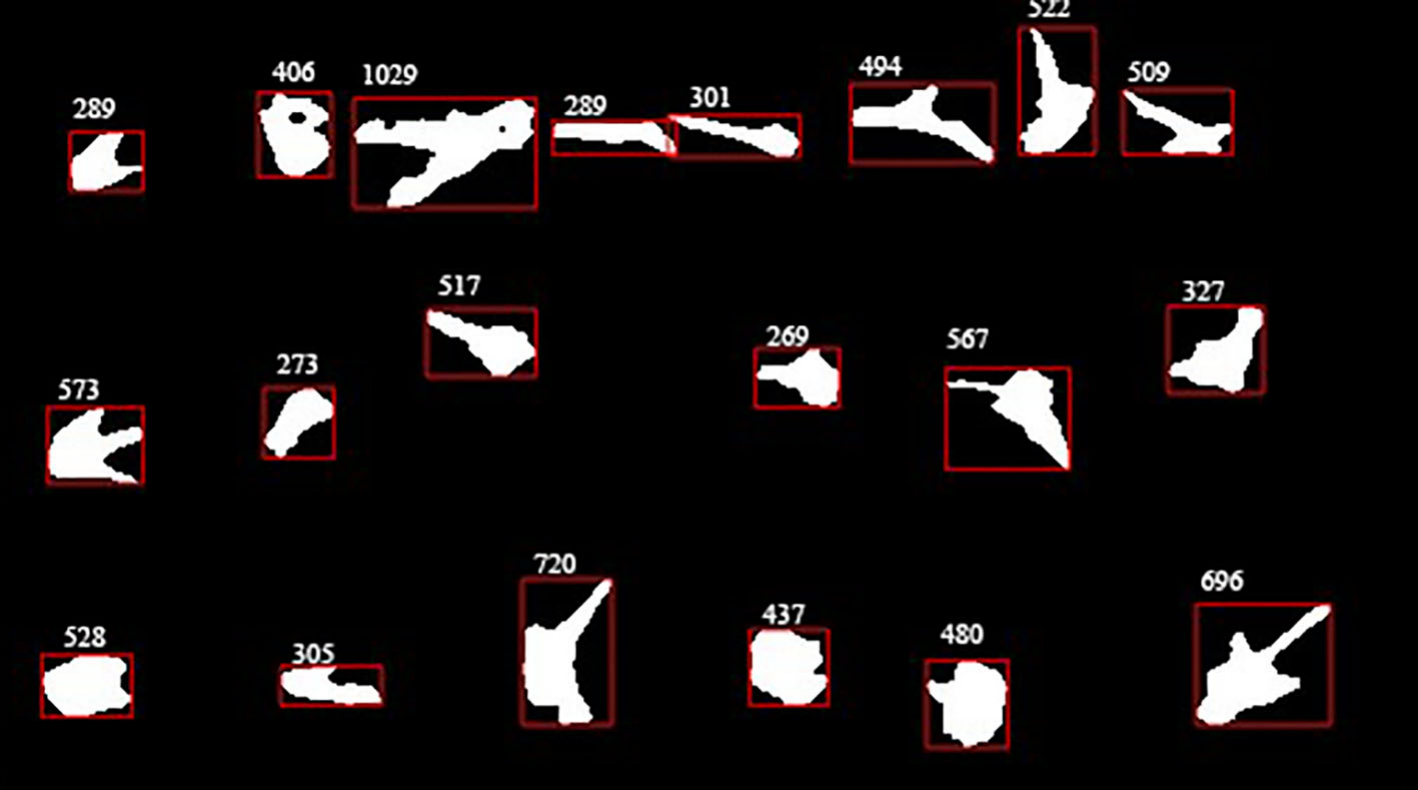
Distance transformation divides the adhesion region.

The rice-type grayscale image obtained through distance transformation shows that the bright core of the foreground area is close to the center of the target, with the edges gradually darkening, indicating that the pixels in the central area are farther from the background and closer to the edges. Overlapping rice areas are usually composed of two independent areas, which appear as two bright cores after distance transformation, with each core representing the center of a rice plant, as shown in **Figure 11**.

After locating the center of the overlapping rice area, different rice areas are identified and separated based on the bright core position. In the grayscale image, 255 represents white, but in the distance transformation image, the brightness of the two cores is different, suggesting that the core brightness of the smaller rice area is lower. Based on the distance transformation results, the centroid position of the rice area is determined, and the overlapping area is split using morphological opening operations. The connected regions are re-detected, and the minimum bounding rectangle is calculated.

### 2.7 Selection of tillering characteristics based on agronomic principles

The tillering stage of rice is the period with the highest nitrogen demand and the fastest growth rate, following the growth pattern of leaves and tillers developing simultaneously. During this stage, the plant’s morphology changes rapidly with the growth of leaves and tillers. Leaf color is an important indicator of the plant’s nutritional status and growth trend: when nitrogen is sufficient, leaves are dark green and tillers are abundant; nitrogen deficiency leads to yellowing leaves and a significant reduction in tillers.

Leaf length during the tillering stage of rice is an important indicator of growth vigor. If adjacent leaves show a significant increase in length and exhibit an outward-extending trend (leading leaves), it indicates vigorous plant metabolism, with a significant expansion of the circumscribed rectangle area; conversely, if leaf length increases minimally and leaves grow in a short, dense cluster (flat leaves), it reflects growth inhibition, with a noticeable reduction in the circumscribed rectangle area. Research indicates that tillering number is significantly correlated with plant morphological characteristics. By analyzing leaf arrangement patterns and outer rectangle parameters, a quantitative relationship model between image features and tillering number can be established.

### 2.8 Branch characteristic extraction

The Swin-Unet rice plant segmentation model was applied to rice canopy images during the tillering stage to achieve plant segmentation, generate mask images, and convert them to binary mode. The pixel area data of rice plants was obtained by extracting white pixels, and the average pixel area of four rice holes in the sample area was ultimately used as the performance indicator for the breeding area. **Figure 10** shows a pixel area extraction diagram of a breeding area.

Based on the segmented single-plant images, morphological features (such as projected area and outer rectangle length and width) and leaf color and shape are extracted to reflect the nutritional status and growth vigor of rice. The number of tillers is estimated through the linear relationship between pixel area and tiller number.

#### 2.8.1 Extraction of rice morphological parameters

##### 2.8.1.1 Rice plant area and perimeter

Convert the segmented RGB rice image into a binary image, count the number of target pixels to calculate the projected area of the plant. Obtain the outer contour of the plant through edge detection, and count the contour pixels to calculate the perimeter.

##### 2.8.1.2 Enclosing rectangle of the plant

The rotation lock algorithm is used to identify the four extreme points (maximum/minimum horizontal and vertical coordinates) of the two-dimensional image of the rice plant, construct the minimum enclosing rectangle, and calculate its height, width, and area. Finally, based on the total projected area obtained, the ratio between the total projected area and the area of the enclosing rectangle is calculated.

##### 2.8.1.3 Fractal dimension

Fractal geometry is used to describe the efficient spatial utilization of complex irregular objects. For irregular two-dimensional rice images, assuming an M×M image, use an ϵ×ϵ square window to cover the foreground points in the binary rice image, and the formula for calculating the fractal dimension is Equation 8:

(8) $$D=\underset{\epsilon \to 0}{lim}\left[logN\left(\epsilon \right)/Log\left(1/\epsilon \right)\right]$$

In the formula, N(ϵ) is the number of ϵ×ϵ squares covering the foreground points in the binary image of the rice plant. When ϵ→0, the fractal dimension D is calculated.

##### 2.8.1.4 Convex hull

Convex hulls are widely used in image processing and pattern recognition and are defined as a combination of exterior segments that enclose the target area. Similar to exterior rectangles, convex hulls can reflect the growth morphology of rice plants. Before extracting the convex hull, it is necessary to first obtain the exterior edge of the target and then extract the convex hull based on the edge.

#### 2.8.2 Extraction of rice plant color parameters

Extract green information from the segmented image: calculate the sum of the gray values of the green region G component and divide it by the total number of pixels to obtain the overall greenness; count the number of green pixels to obtain the green projection area; calculate the proportion of green pixels in the entire image to obtain the green ratio.

### 2.9 Selection of tillering characteristics

#### 2.9.1 Normalization of tillering characteristic data

The rice tiller characteristics extracted from UAV images are divided into morphological features and color features, totaling 12 items: the 10 morphological features include Total Area, Perimeter, Bounding Rectangle Height, Bounding Rectangle Width, Area to Rectangle Area Ratio, Rectangle Aspect Ratio, Fractal Dimension, Perimeter to Area Ratio, Convex Hull Area, and Area to Convex Hull Area Ratio, while the 2 color features are Green Value (i.e., the average gray value of the G component in the green area) and Green Ratio (i.e., the proportion of green pixels in the total pixels of the image).In rice feature analysis, different dimensions and units of parameters, such as color and shape, may cause errors. To eliminate this influence, a normalization method is used to map the data to a range of 1 to 2, thereby improving model accuracy and reducing the influence of extreme values.

#### 2.9.2 Correlation analysis of tillering characteristics

**Figure 12:**
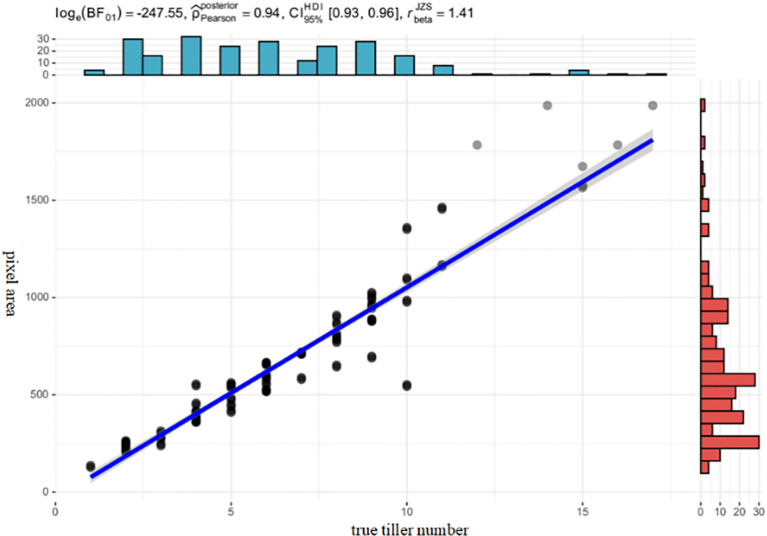
Correlation analysis between pixel area and true tiller number.

Image data collected by a drone at a height of 12 meters was used to crop and manually segment the tillering area, extract rice plant characteristics, and verify the correlation between pixel area and actual tillering number through Bayesian statistical analysis (**Figure 12**).

**Figure 13:**
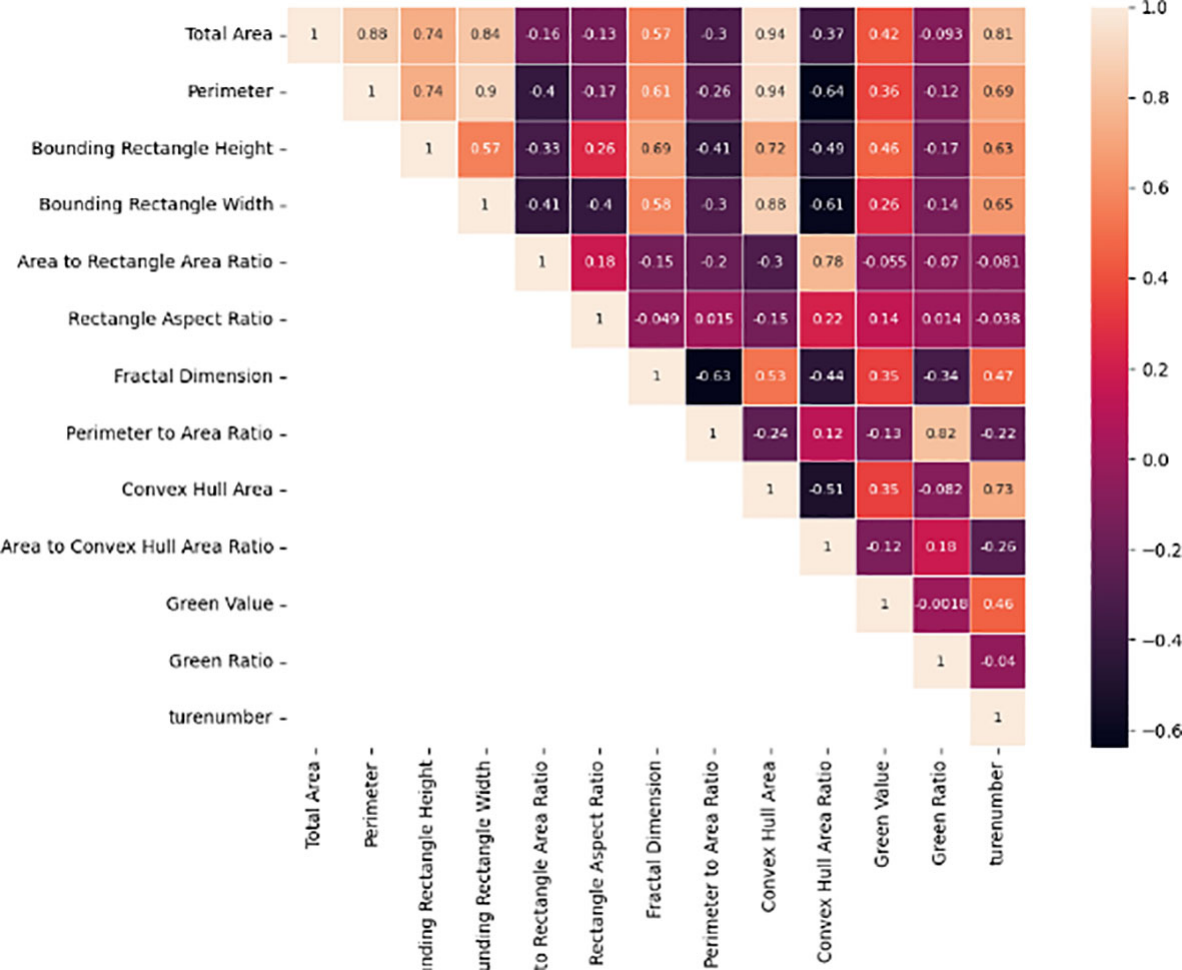
Correlation between color morphological character is tics and tillers(the horizontal and vertical coordinates are image features and tillers).

The blue rectangle above represents the histogram of the actual tiller count statistics, while the red rectangle on the right represents the histogram of pixel area statistics. The Bayesian statistical method was selected, with the correlation metrics being the logarithmic values of the Bayesian factors, the Pearson correlation coefficient, and the 95% confidence interval. The Bayesian analysis framework using the JZS prior was employed to measure the effect of the regression coefficient (β). The analysis shows that the correlation between pixel area and tiller number in 230 rice images reaches 94%, with the Pearson coefficient approaching 1, indicating a strong correlation between the two. Therefore, pixel area can be used as the independent variable in the regression model to estimate the number of tillers. After analyzing the correlation between pixel area and tiller number, we further analyzed the correlation between all features and tiller number and plotted a correlation matrix diagram (**Figure 13**).

Among the 10 morphological characteristics and 2 color characteristics, 7 are positively correlated with the number of tillers. The highest correlation was observed for pixel area, with a correlation coefficient of 0.81. Morphological features such as perimeter, length, and width of the bounding rectangle had correlation coefficients close to 0.65 with tillering number, while among color features, only greenness was correlated with tillering number, with a correlation coefficient of 0.46. The results indicate that changes in tillering number during the tillering stage of rice are complex, and a single feature is insufficient to comprehensively reflect the entire change process.

## 3 Results

Using the selected feature data, we established a tillering number estimation model using traditional linear regression and forest regression, support vector regression, XGBoost, and other algorithms, and selected the optimal model through comparative analysis. The algorithms were implemented using the Python Scikit-learn library. We randomly selected 80% of the data (1,790 actual tiller count measurements) as the training set and the remaining 20% (450 actual tiller count measurements) as the test set to evaluate model performance.

### 3.1 Single-factor linear regression model construction

From the results of the correlation analysis of tillering characteristics, it can be seen that the correlation coefficients between morphological characteristics and tillering numbers are relatively high. However, overall, the changes in tillering numbers are complex. To verify the effectiveness of the single-factor linear regression model, corresponding tillering number estimation models were established for the top five morphological characteristics and one color characteristic, as shown in **Table 3**.

**Table 3:** Unary and multiple-factor linear regression tiller number estimation models and validation indices.

ID	Argument	Model	RMSE	R^2^	MAE	NRMSE
F1	Plant area	y=0.0045x+3.5401	0.67	0.6408	0.59	0.11
F2	Convex area	y=(x+941.22)/338.57	0.71	0.5257	0.63	0.18
F3	Perimeter	y=(x+6.0853)/32.942	0.95	0.4827	0.64	0.19
F4	Width of the circumscribed rectangle	y=(x-8.4814)/5.6729	1.33	0.4237	0.43	0.22
F5	Height of the circumscribed rectangle	y=(x-14.771)/4.0525	1.49	0.4002	0.64	0.23
F6	Greenness	y=(x-11.172)/0.883	1.54	0.2095	0.66	0.36
MF1	Total Area, Perimeter	y=3.6759+(0.0048*x1)+(-0.0017*x2)	0.66	0.6212	0.62	0.12
MF2	Total Area, Perimeter, Convex Hull Area	y=2.9822+(0.0062*x1)+(0.0036*x2r)+(-0.0011*x3)	0.59	0.6218	0.60	0.11
MF3	Total Area, Bounding Rectangle Height, Convex Hull Area, Green Value	y=2.0999+(0.0057*x1)+(0.0150*x2)+(-0.0006*x3+(0.0405*x4)	0.57	0.7542	0.53	0.11
MF4	Total Area, Perimeter, Bounding Rectangle Height, Convex Hull Area, Green Value	y=2.4355+(0.0063*x1)+(0.0025*x2)+(-0.0097*x3)+(-0.0008*x4)+(0.0434*x5)	0.67	0.6825	0.63	0.12
MF5	Total Area, Perimeter, Bounding Rectangle Width, Bounding Rectangle Height, Convex Hull Area, Green Value	y=2.1262+(0.0058*x1)+(0.0008*x2)+(-0.0024*x3)+(0.0138*x4)+(-0.0007*x5)+(0.0394*x6)	0.67	0.6529	0.61	0.12

y is the number of tillers, x is the independent variable, and the F1-6model error is sorted from low to high, the same as below. Total Projected Area, Perimeter, Height of the Bounding Rectangle, Width of the Bounding Rectangle, Ratio of Total Projected Area to Rectangle Area, Aspect Ratio of the Rectangle, Fractal Dimension of the Binary Image, Perimeter to Area Ratio, Convex Hull Area, Area to Convex Hull Area Ratio, Green Value, Green Ratio.

### 3.2 Construction of a multi-factor linear regression model

Based on feature correlation analysis, the six feature indicators with the highest correlation were selected, all possible feature combinations were generated, and a tillering number estimation model was constructed based on these combinations. The results of the multiple linear regression model and validation set evaluation are shown in **Table 4**.

**Table 4:** Machine learning model estimation accuracy.

Machine learning methods	Total number of features	R^2^ Training	Testing	RMSE Training	Testing	NRMSE Training	Testing
RFR	12	0.7512	0.7438	0.5715	0.6007	0.1212	0.1347
SVR	12	0.7617	0.7435	0.5804	0.5997	0.1018	0.1127
XGBoost	12	0.8267	0.8021	0.5760	0.5778	0.1014	0.1125
PSO-RFR	12	0.8014	0.7984	0.4412	0.4217	0.0987	0.1014
PSO-SVR	12	0.8289	0.8037	0.4718	0.4916	0.1036	0.1124
PSO-XGBoost	12	0.8507	0.8423	0.3328	0.3459	0.0878	0.0993

### 3.3 Machine learning-based model building

Based on features extracted from visible light RGB images captured by drones, we constructed a tillering number estimation model using random forest regression (RFR), support vector regression (SVR), and extreme gradient boosting (XGBoost) algorithms. The results are shown in **Table 5**.We then optimized the model parameters using the particle swarm optimization (PSO) algorithm to improve estimation accuracy.

**Table 5:** Comparison of different rice tiller number estimation models.

Estimation model	R² (training/testing)	RMSE (training/testing)	NRMSE (training/testing)	Note
Single factor (pixel area)	0.6408/-	0.67/-	0.12/-	Significant increase in error at tiller number >10
Multi-factor (combination of 4 features)	0.7542/-	0.57/-	0.11/-	Accuracy no longer improves for features > 4
Random Forest (RFR)	0.7512/0.7438	0.5715/0.6007	0.1212/0.1347	General adaptability to nonlinear relationships
Support Vector Regression (SVR)	0.7617/0.7435	0.5804/0.5997	0.1018/0.1127	Robust
XGBoost	0.8267/0.8021	0.5760/0.5778	0.1014/0.1125	Best performance in unoptimized model
PSO-RFR	0.8014/0.7984	0.4412/0.4217	0.0987/0.1014	About 30% error reduction after optimization
PSO-SVR	0.8289/0.8037	0.4718/0.4916	0.1036/0.1124	Limited optimization effect
PSO-XGBoost	0.8507/0.8423	0.3328/0.3459	0.0878/0.0993	Optimal model (85% accuracy)

## 4 Discussion

### 4.1 Visualization analysis of rice plant segmentation model

**Figure 14:**
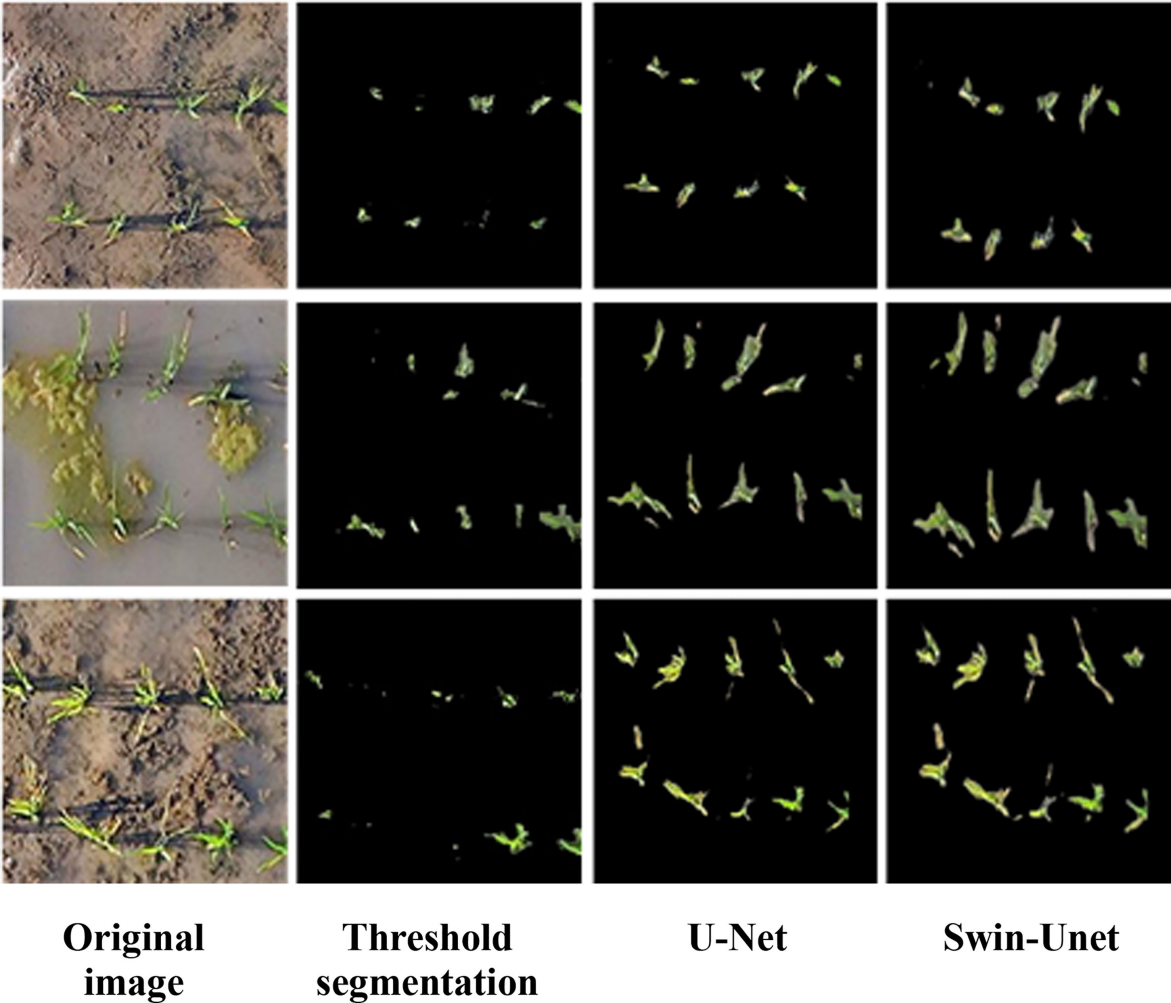
Segmentation result.

To validate the performance of the image segmentation network, we conducted a visual comparison analysis of the results obtained using OTSU threshold segmentation, the U-Net algorithm, and Swin-Unet segmentation of rice plants. From the segmentation results of the test set, we selected three images affected by small objects, algae weeds, and yellowed leaves, and the segmentation results are shown in the **Figure 14**.

As shown in the visualization results, Swin-Unet demonstrates a significant advantage, with segmentation performance that outperforms other algorithms. As can be seen from **Figure 14**, the U-Net network can divide the entire rice image into regions closer to the true labels and reduce the error partitioning of traditional segmentation algorithms. However, the U-Net model still has some issues in identifying the boundary details of rice plants and lacks sufficient accuracy in recognizing the edges of rice plants, resulting in some differences from the true labels. This is related to the insufficient extraction of shallow features in the encoder part of the U-Net model. Although the U-Net model has integrated shallow and deep features through skip connections, the integrated shallow features are still insufficient, leading to difficulties in accurately segmenting the edges of rice plants. The method proposed in this paper achieves results that are highly consistent with the ground truth labels, both in terms of edge details and overall shape, with clear and detailed boundary contours, the most superior segmentation performance, and insensitivity to the surrounding environment, enabling effective segmentation of rice plants.

### 4.2 Comparative analysis of rice tillering number estimation models

By comparing traditional linear regression, machine learning regression algorithms, and their particle swarm optimization (PSO) versions, the performance of the models in estimating tillering numbers was evaluated. Key metrics included the coefficient of determination (R²), root mean square error (RMSE), and normalized root mean square error (NRMSE). The results are shown in **Table 5**.

By comparing the performance of traditional linear regression and machine learning models in rice tiller number estimation, it was found that although the traditional linear model (e.g., multifactor regression R² = 0.7542) outperformed the one-factor model (R² = 0.6408), its linear assumption led to significant errors when the tiller number was higher; whereas, in the machine learning model, the accuracy of the unoptimized test set of XGBoost reached R² = 0.8021. After particle swarm optimization (PSO), the test set R² of PSO-XGBoost was further improved to 0.8423, the RMSE was reduced by 38% to 0.3459, and the estimation accuracy reached 85%, which made it the optimal model. The results indicate that PSO-XGBoost can effectively support complex tiller number estimation by virtue of its nonlinear fitting and parameter optimization advantages, while the traditional model is suitable for data-limited scenarios but needs to be wary of high value errors. This study provides a high-precision method for the assessment of tiller status in precision agriculture, and the generalizability of the model can be further optimized by combining multi-source remote sensing data in the future.

### 4.3 Field application and thematic mapping of tiller number estimation

The PSO-XGBoost rice tiller number estimation model was applied to validate the application of tiller number in breeding plots. The experiment randomly selected 576 breeding plots that did not participate in the model training in 2024, and the digital images at the early stage of tillering were used for the estimation of tiller number and thematic mapping, which in turn provided theoretical support for the identification of tillering traits in breeding materials.

Rice orthophotos were synthesized using DJI Terra software, and the latitude and longitude positioning data of the visible images were obtained through ArcGIS software. The checkerboard segmentation technique was used to divide the image into equal rectangular regions according to preset scale parameters, which ignores image contours and has fast computing speed, and the scale parameter is the only adjustment parameter to determine the grid size.

The results of tiller number estimated by PSO-XGBoost model prediction were spatially connected with grid cell IDs to generate an attribute table containing geocodes. The number of tillers was classified into five classes in ArcGIS software using the Jenks optimization method: low tiller area: ≤83 plants/area, medium-low tiller area: 84–114 plants/area, medium tiller area: 115–143 plants/area, medium-high tiller area: 144–171 plants/area, and high tiller area: ≥172 plants/area (3 rows per plot*)./Zone (3 rows * 9 holes = 27 holes per plot, i.e., the number of tillers per hole ranges from 2 to 9).

**Figure 15:**
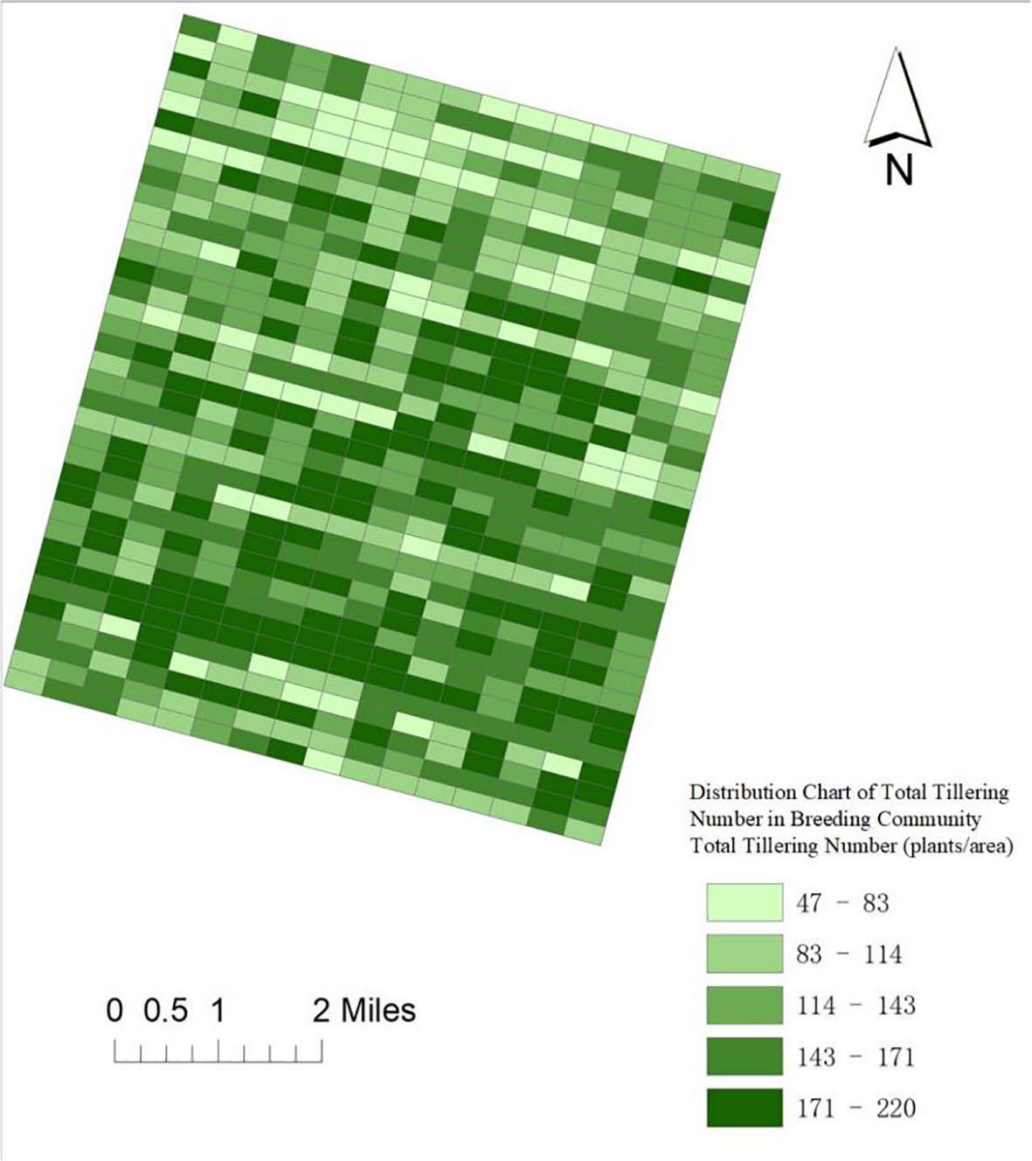
Spatial visualization distribution of tiller number estimation.

In order to enhance the visualization effect, the gradient green color (RGB 0-255: 102-255–102 to 0-153-0) was used to symbolize the different tiller classes (**Figure 15**).

## 5 Conclusion

1. Aiming at the defects of traditional image segmentation methods (cumbersome process, slow speed, high sensitivity to shooting angle/lighting, and poor robustness) in rice canopy image processing, this study proposes a targeted solution. First, data preprocessing (combining histogram equalization and filtering denoising) is performed to solve the problems of low image contrast, blurred rice-background boundaries, high-frequency noise, edge distortion, and visual interference caused by variable lighting (see **Figure 5** for effects). Then, a Swin-UNet segmentation model is constructed by integrating Swin Transformer into U-Net: the symmetric structure of U-Net is retained, and Swin Transformer is introduced in the encoder to improve the classification accuracy of rice boundaries in UAV digital images; sub-pixel convolution is used to optimize the decoder’s up-sampling structure, which fuses with encoder features to enhance small-target recognition. Finally, post-processing (connected-domain labelling + distance transformation + cyclic threshold segmentation) resolves local adhesion and noise interference. The model achieves an average segmentation accuracy of 92.5% (7.2% higher than U-Net), providing high-quality image foundation for subsequent tiller feature extraction and realizing efficient and accurate segmentation of rice plants under complex backgrounds.
2. Combining the agronomic mechanism of rice tillering with morphological and color feature screening, this study clarifies the key indicators related to tillering ability. It is confirmed that leaf area is closely associated with tillering potential, and leaf color can reflect plant nutritional status and growth status. A total of 12 tiller-related features are extracted, including 10 morphological features (plant area, outer rectangle height/width, perimeter, convex hull area, biplot fractal dimension, etc.) and 2 color features (greenness, green percentage). Correlation analysis further clarifies the correlation degree between each feature and tiller number, providing accurate and scientifically supported feature data for subsequent tiller number estimation and laying a foundation for the rationality of the estimation model.
3. To improve the accuracy of tiller number estimation, the XGBoost algorithm is optimized by particle swarm optimization (PSO), and a tiller number estimation model is built by integrating the 10 morphological and 2 color features with rice tillering agronomic mechanism. Experimental results show that the PSO-XGBoost model achieves R²=0.85 and RMSE = 0.35, outperforming traditional linear regression and other machine learning algorithms due to its advantages in nonlinear fitting and parameter optimization. Furthermore, the model is applied to 576 breeding plots at the early tillering stage captured by UAV in 2024: the checkerboard segmentation technique and natural breakpoint classification method are used to divide tiller number into 5 grades, realizing visual distinction of different tillering levels. This provides quantitative technical support for auxiliary identification of rice tillering traits, and has practical application value for accelerating rice breeding process and improving breeding efficiency.

## Data Availability

The original contributions presented in the study are included in the article/supplementary material. Further inquiries can be directed to the corresponding author.

## References

[B1] Cao H. Wang Y. Chen J. Jiang D. Zhang X. Tian Q. . (2022). “ Swin-unet: Unet-like pure transformer for medical image segmentation,” in European conference on computer vision (Cham, Switzerland: Springer Nature).

[B2] Cao Y. L. Zhang H. Z. Guo F. X. Feng S. Yang L. L. Wei S. H. . (2024). Research progress of crop disease monitoring based on UAV remote sensing. J. Shenyang Agric. Univ. 55, 616–628. doi: 10.3969/j.issn.1000-1700.2024.05.012, PMID: 35900448

[B3] Cao Z. S. Li Y. D. Ye C. Shu S. F. Sun B. F. Huang J. B. . (2020). Model for monitoring tiller number of double cropping rice based on hyperspectral reflectance. Trans. Chin. Soc. Agric. Eng. 36, 185–192. doi: 10.11975/j.issn.1002-6819.2020.04.022

[B4] Chua L. O. (1997). CNN: A vision of complexity. Int. J. Bifurcation Chaos. 7, 2219–2425. doi: 10.1142/S0218127497001618, PMID: 40951326

[B5] Du M. M. Roshanianfard A. Liu Y. C. (2021). Inversion of wheat tiller density based on visible-band images of drone. Spectrosc. Spectral Anal. 41, 3828–3836. doi: 10.3964/j.issn.1000-0593(2021)12-3828-09, PMID: 41774970

[B6] Fan M. Y. Ma Q. Liu J. M. Wang Q. Wang Y. Duan X. C. . (2015). Counting method of wheatear in field based on machine vision technology. Trans. Chin. Soc. Agric. Machinery. 46, 234–239. doi: 10.6041/j.issn.1000-1298.2015.S0.038

[B7] Fan Y. B. Wu S. R. Kuang W. Chen Y. X. Fang B. H. Ren J. Q. . (2025). Estimation of nitrogen content in rice grains based on UAV hyperspectral imagery. Trans. Chin. Soc. Agric. Machinery. 56, 332–343 + 423. doi: 10.6041/j.issn.1000-1298.2025.01.032

[B8] Hayat M. A. Wu J. X. Cao Y. L. (2020). Unsupervised Bayesian learning for rice panicle segmentation with UAV images. Plant Methods. 16. doi: 10.1186/s13007-020-00567-8, PMID: 32123536 PMC7035759

[B9] Jiang K. L. (2022). Research on inversion method of chlorophyll content in rice canopy leaves based on UAV hyperspectral remote sensing ( Shenyang Agricultural University). doi: 10.27327/d.cnki.gshnu.2022.000185

[B10] Li J. P. Feng S. Yang X. Li G. M. Zhao D. X. Yu F. H. . (2023). Unsupervised extraction of rice coverage with incorporating CLAHE-SV enhanced Lab color features. Trans. Chin. Soc. Agric. Eng. 39, 195–206. doi: 10.11975/j.issn.1002-6819.202305119

[B11] Li X. Qian Q. Fu Z. . (2003). Control of tillering in rice. Nature. 422, 618–621. doi: 10.1038/nature01518, PMID: 12687001

[B12] Li P. L. Zhang X. Wang W. H. Zheng H. B. Yao X. . (2021). Research on rice yield monitoring based on hyperspectral and LiDAR remote sensing. Scientia Agricultura Sin. 54, 2965–2976.

[B13] Liang W. Shang F. Lin Q. Lou C. Zhang J. . (2014). Tillering and panicle branching genes in rice. Gene 537, 1–5. doi: 10.1016/j.gene.2013.11.058, PMID: 24345551

[B14] Liu G. Y. Liu P. Y. Wei W. J. Zhang S. Y. Li H. T. . (2014). Method of image segmentation for touching maize kernels. Trans. Chin. Soc. Agric. Machinery. 45, 285–290. doi: 10.6041/j.issn.1000-1298.2014.09.046

[B15] Liu Z. Y. Zhou Y. C. Liang C. W. Li R. Y. Zhang Y. . (2025). Method of counting rice grains in ears based on deformable convolution. Trans. Chin. Soc. Agric. Machinery. 56, 363–373. doi: 10.6041/j.issn.1000-1298.2025.03.036

[B16] Lottes P. Behley J. Chebrolu N. Milioto A. Stachniss C. . (2020). Robust joint stem detection and crop-weed classification using image sequences for plant-specific treatment in precision farming. J. Field Robotics. 37, 20–34. doi: 10.1002/rob.21901, PMID: 41773552

[B17] Ninomiya S. (2022). High-throughput field crop phenotyping: current status and challenges. Breed. Sci. 72, 3–18. doi: 10.1270/jsbbs.21069, PMID: 36045897 PMC8987842

[B18] Pei H. J. Feng H. K. Li C. C. Jin X. L. Li Z. H. Yang G. J. . (2017). Remote sensing monitoring of winter wheat growth with UAV based on comprehensive index. Trans. Chin. Soc. Agric. Eng. 33, 74–82. doi: 10.11975/j.issn.1002-6819.2017.20.010

[B19] Pipatsitee P. Eiumnoh A. Tisarum R. Taota K. Kongpugdee S. Sakulleerungroj K. . (2020). Above-ground vegetation indices and yield attributes of rice crop using unmanned aerial vehicle combined with ground truth measurements. Notulae Botanicae Horti Agrobotanici Cluj-Napoca. 48, 2385–2398.

[B20] Qi H. Sun H. F. Lv L. J. Li S. Min J. N. Hou L. . (2025). Wheat leaf area index estimation based on fusion of UAV multispectral information and exture features. Trans. Chin. Soc. Agric. Machinery. 56, 334–344. doi: 10.6041/j.issn.1000-1298.2025.03.033

[B21] Roth L. Camenzind M. Aasen H. Kronenberg L. Barendregt C. Camp K.-H. . (2020). Repeated multiview imaging for estimating seedling tiller counts of wheat genotypesUsing drones. Plant Phenomics. doi: 10.34133/2020/3729715, PMID: 33313553 PMC7706335

[B22] Sun Y. Guo H. R. Chen X. A. Li M. Q. Fang B. Cao Y. L. (2025a). YOLOv8n-SSDW: A lightweight and accurate model for barnyard grass detection in fields. Agriculture. 15, 1510. doi: 10.3390/agriculture15141510, PMID: 41725453

[B23] Sun Y. Li M. Liu M. Zhang J. Cao Y. Ao X. . (2025b). A statistical method for high-throughput emergence rate calculation for soybean breeding plots based on field phenotypic characteristics. Plant Methods. 21, 40. doi: 10.1186/s13007-025-01356-x, PMID: 40122826 PMC11931824

[B24] Takai T. (2024). Potential of rice tillering for sustainable food production. J. Exp. Bot. 75, 708–720. doi: 10.1093/jxb/erad422, PMID: 37933683 PMC10837021

[B25] Tao H. L. Xv L. J. Feng H. K. Yang G. J. Dai Y. Niu Y. C. . (2020). Estimation of plant height and leaf area index of winter wheat based on UAV hyperspectral remote sensing. Trans. Chin. Soc. Agric. Machinery. 51, 193–201. doi: 10.6041/j.issn.1000-1298.2020.12.02

[B26] Wan L. Cen H. Y. Zhu J. P. Zhang J. F. Du X. Y. He Y. . (2020). Using fusion of texture features and vegetation indices from water concentration in rice crop to UAV remote sensing monitor. Smart Agric. 2, 58–67. doi: 10.12133/j.smartag.2020.2.1.201911-SA002

[B27] Wang X. H. Li X. M. Tang Q. Y. Zou D. Luo Y. Y. Li K. F. . (2024). Construction and application of dynamic tillering model for rice population. Trans. Chin. Soc. Agric. Eng. 40, 213–221. doi: 10.11975/j.issn.1002-6819.202309173

[B28] Wang B. Zhang Z. (2020) in Green crop image segmentation based on superpixel blocks and decision tree. 2020, 3–17, Sun X. doi: 10.1007/978-3-030-57881-7_1, PMID: 41773268

[B29] Wu J. L. Wu H. Q. Li H. Lei X. P. Song H. Y. . (2024). Segmentation of buckwheat by UAV based on improved lightweight deepLabV3+ at seedling stage. Trans. Chin. Soc. Agric. Machinery. 55, 186–195. doi: 10.6041/j.issn.1000-1298.2024.05.017

[B30] Wu Y. L. Zhao L. Jiang H. Y. Guo X. Q. Huang F. . (2014). Image segmentation method for green crops using improved mean shift. Trans. Chin. Soc. Agric. Eng. 30, 161–167. doi: 10.3969/j.issn.1002-6819.2014.24.019, PMID: 35900448

[B31] Xiong X. Lingfeng D. Lingbo L. Haifu T. Peng Y. Dan W. . (2017). Panicle-SEG: a robust image segmentation method for rice panicles in the field based on deep learning and superpixel optimization. Plant Methods. 13, 104. doi: 10.1186/s13007-017-0254-7, PMID: 29209408 PMC5704426

[B32] Yu F. Bai J. Fang J. Sien G. Zhu S. Xu T. (2024). Integration of a parameter combination discriminator improves the accuracy of chlorophyll inversion from spectral imaging of rice. Agric. Commun. 2, 100055. doi: 10.1016/j.agrcom.2024.100055, PMID: 41774992

[B33] Yue J. Li T. Feng H. Fu Y. Liu Y. Tian J. . (2024). Enhancing field soil moisture content monitoring using laboratory-based soil spectral measurements and radiative transfer models. Agric. Commun. 2, 100060.

[B34] Zhu W. J. Dai S. Y. Feng Z. K. Duan K. W. Shao C. F. Wei X. H. . (2024). Estimation of rice basic seedling number based on mixed pixel decomposition. Trans. Chin. Soc. Agric. Machinery. 55, 202–209. doi: 10.6041/j.issn.1000-1298.2024.06.021

[B35] Zhu Q. Z. Qiu Y. Q. Wang A. C. Zhang L. Y. . (2024). Accurate inversion of rice chlorophyll content by integrating multispectral and texture features derived from UAV multispectral lmagery. Trans. Chin. Soc. Agric. Machinery. 55, 287–293. doi: 10.6041/j.issn.1000-1298.2024.12.027

[B36] Zhu L. S. Xu M. Y. Wang A. C. Zhang D. F. Qian W. H. Li C. . (2023). Research on rice growth density detection at maturity stage based on improved DeepLabV3+. J. Chin. Agric. Mechanization. 44, 186–192. doi: 10.13733/j.jcam.issn.2095-5553.2023.12.028

